# Ras drives malignancy through stem cell crosstalk with the microenvironment

**DOI:** 10.1038/s41586-022-05475-6

**Published:** 2022-11-30

**Authors:** Shaopeng Yuan, Katherine S. Stewart, Yihao Yang, Merve Deniz Abdusselamoglu, S. Martina Parigi, Tamar Y. Feinberg, Karen Tumaneng, Hanseul Yang, John M. Levorse, Lisa Polak, David Ng, Elaine Fuchs

**Affiliations:** 1 Robin Chemers Neustein Laboratory of Mammalian Cell Biology and Development, The Rockefeller University, New York, NY USA; 2 Howard Hughes Medical Institute, New York, NY USA; 3 Present Address: Volastra Therapeutics, New York, NY USA; 4 Present Address: Sanofi, Cambridge, MA USA; 5 Present Address: Department of Biological Sciences, Korea Advanced Institute of Science and Technology, Daejeon, Korea; 6 Present Address: Temple University, Philadelphia, PA USA

**Keywords:** Cancer microenvironment, Tumour angiogenesis, Cancer stem cells, Squamous cell carcinoma, Cell signalling

## Abstract

Squamous cell carcinomas are triggered by marked elevation of RAS–MAPK signalling and progression from benign papilloma to invasive malignancy^1–4^. At tumour–stromal interfaces, a subset of tumour-initiating progenitors, the cancer stem cells, obtain increased resistance to chemotherapy and immunotherapy along this pathway^5,6^. The distribution and changes in cancer stem cells during progression from a benign state to invasive squamous cell carcinoma remain unclear. Here we show in mice that, after oncogenic RAS activation, cancer stem cells rewire their gene expression program and trigger self-propelling, aberrant signalling crosstalk with their tissue microenvironment that drives their malignant progression. The non-genetic, dynamic cascade of intercellular exchanges involves downstream pathways that are often mutated in advanced metastatic squamous cell carcinomas with high mutational burden^7^. Coupling our clonal skin *HRAS*^*G12V*^ mouse model with single-cell transcriptomics, chromatin landscaping, lentiviral reporters and lineage tracing, we show that aberrant crosstalk between cancer stem cells and their microenvironment triggers angiogenesis and TGFβ signalling, creating conditions that are conducive for hijacking leptin and leptin receptor signalling, which in turn launches downstream phosphoinositide 3-kinase (PI3K)–AKT–mTOR signalling during the benign-to-malignant transition. By functionally examining each step in this pathway, we reveal how dynamic temporal crosstalk with the microenvironment orchestrated by the stem cells profoundly fuels this path to malignancy. These insights suggest broad implications for cancer therapeutics.

## Abstract (web summary)

Aberrant crosstalk between cancer stem cells and their microenvironment triggers angiogenesis and TGFβ signalling, creating conditions that are conducive for hijacking leptin and leptin receptor signalling, which in turn launches downstream PI3K–AKT–mTOR signalling during the benign-to-malignant transition.

## Main

Squamous cell carcinomas (SCCs) are common life-threatening cancers of the stratified epithelia of skin, oral cavity, oesophagus and lungs^1,8,9^. Even for skin, where SCCs are often caught early, their frequency of occurrence and ever-rising metastatic incidences pose major health concerns^10^. Chemical carcinogenesis studies expose elevated RAS–MAPK signalling, often involving oncogenic *Ras* mutations, as critical in the path to invasive SCCs^2–4^. The lengthy delay and sporadic nature of mutagen-mediated SCCs has led to the view that additional oncogenic mutations are needed^3,11–13^, further supported by the high mutational burden associated with human metastatic SCCs^7^. However, genetically induced SCCs display many fewer mutations than mutagen-driven SCCs^3,14^, and skin tumours exhibiting a heterogeneous benign/SCC phenotype can be initiated even with *HRAS*^*G12V*^ alone^6^. These observations raise the possibility that non-genetic alterations may be potent cancer drivers.

Increasing evidence has highlighted extrinsic perturbations—for example, inflammation, metabolism and wounding—in preconditioning tissues to heightened cancer vulnerabilities^6,14–19^. It is less clear whether and how in healthy tissues an oncogenic mutation in a stem cell can intrinsically stimulate environmental changes that may lessen the need for multi-step mutagenesis. Here we address this issue using a single *HRAS*^*G12V*^ oncogene model that clonally activates a reliable path to aggressive, invasive cutaneous SCCs. After performing deep single-cell RNA sequencing (scRNA-seq) to gain insights into the SCC cancer stem cell (CSC) signature, we trace its temporal origins and physiological importance. We show that, after oncogenic RAS initiation, tissue stem cells begin an aberrant molecular dialogue with their surroundings, culminating in a considerable remodelling of the tumour microenvironment at the benign-to-malignant transition. This provides fertile ground for stromal TGFβ-mediated induction of leptin receptor (*Lepr*) and vasculature-mediated elevation of tissue leptin, leading to LEPR–leptin signalling and PI3K–AKT–mTOR in CSCs to drive the invasive switch. Triggered by oncogenic RAS, each step of this stem cell–microenvironment crosstalk cascade is essential for malignant progression, and it involves pathways that are often mutated in advanced SCCs with a high mutational burden.

## Newfound heterogeneity in CSCs

Skin stem cells that acquire *HRAS* mutations go through a benign papilloma state before progressing to malignant, invasive SCCs^20,21^. On the basis of serial transplantations, tumour-initiating CSCs from mouse SCCs are enriched for integrins and reside at tumour-stromal interfaces^22,23^. In tumours displaying a mixed phenotype, basal progenitors undergoing TGFβ signalling are enriched for CSCs with increased resistance to chemotherapy and immunotherapy, and the loss of TGFβ signalling reverts tumours to a benign state^5,6,19^.

**Fig. 1:**
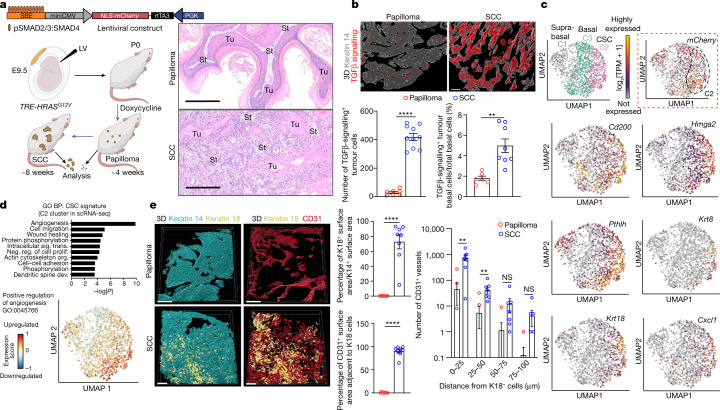
Benign-to-invasive rewiring of the tumour-initiating CSC transcriptome fuels angiogenesis. **a**, The tumour model. Lentivirus containing a TGFβ mCherry reporter and transactivator rtTA3 was injected at a low titre in utero into the amniotic sacs of E9.5 *TRE-HRAS*^*G12V*^ mouse embryos to sparsely transduce individual skin progenitors. Postnatally, doxycycline activates rtTA3 and induces *HRAS*^*G12V*^ in these stem cells. Haematoxylin and eosin (H&E) staining reveals temporally distinct pathologies of benign and malignant SCCs. Tu, tumour; St, stroma. Scale bars, 300 µm. **b**, Quantification of a collapsed *z*-stack of 3D whole-mount immunofluorescence images and FACS-purified mCherry^+^ITGA6^high^ basal progenitors reveals increased TGFβ signalling as tumours progress to invasive SCCs (Extended Data Fig. 1b). Bottom left: *n* = 7 (papilloma) and *n* = 10 (SCC); bottom right: *n* = 6 (papilloma) and *n* = 8 (SCC) tumours per stage. *P* *<* 0.0001 (left) and *P* = 0.0018 (right). Scale bars, 50 µm. **c**, UMAP representations and unsupervised *k*-nearest-neighbour-based clustering of single-cell transcriptomes performed on pooled FACS-isolated integrin^low^ (spiked, 159 total suprabasal) and integrin^high^ (bulk, 1,346 total basal) cells from invasive SCC tumours. Clusters C2 and C3, basal progenitors; C1, suprabasal cells. Note that *mCherry* (TGFβ reporter, dotted box) is enriched in, but not exclusive to, C2 (35.8% of all basal cell progenitors). C2 is enriched for markers of SCC-CSCs with tumour-initiating and invasive properties. The UMAP plots show the relative expression levels (log_2_[TPM + 1]) of these genes across single cells. See also Extended Data Fig. 1g. **d**, Angiogenesis is the top GO biological process (BP) term of C2 CSC transcripts (UMAP displays clustering). *P* values were calculated using DAVID bioinformatic analysis. See also Extended Data Fig. 3. Dev., development; neg. reg., negative regulation; org., organization; prolif., proliferation; sig. trans., signal transduction. **e**, 3D collapsed whole-mount immunofluorescence images of the invasive fronts of tissue sections. Keratin 18 (K18) identifies CSCs; CD31 identifies vasculature. Scale bars, 150 µm. Quantifications are of keratin 18^+^ cell abundance, proximity to vessels and distances with vessels. *n* = 8 (top middle), *n* = 8 (bottom middle) and *n* = 8 (right) tumours per condition per stage. *P* < 0.0001 (top and bottom middle); and *P*_0–25_ = 0.0020, *P*_25–50_ = 0.0176, *P*_50–75_ = 0.1337, *P*_75–100_ = 0.1358. For **b** and **e**, statistical analysis was performed using unpaired two-tailed Student’s *t*-tests; NS, *P* ≥ 0.05; *P ≤ 0.05, **P ≤ 0.01, ***P ≤ 0.001, ****P ≤ 0.0001. Data are mean ± s.e.m. (**b** and **e**). See also Supplementary Tables 1–3. The diagram in **a** was created using BioRender. Source data

**Extended Data Fig. 1:**
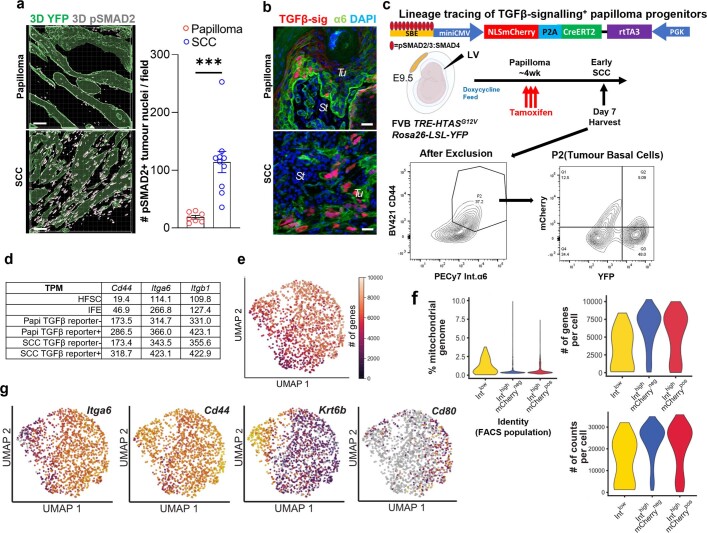
Benign papillomas and invasive SCCs exhibit distinct molecular signatures for angiogenesis and TGFβ responsiveness. **a**, Collapsed z-stack rendering of 3D whole mount immunofluorescence for nuclear pSMAD2. Scale bars, 50 µm. (*n* = 8 tumours per stage, *p* = 0.0007). **b**, Immunofluorescence of sagittal tumour sections for TGFβ reporter (mCherry) signalling, α6 integrin to demarcate the tumour-stromal border and DAPI (nuclei). Note elevated mCherry at invasive fronts of the SCC (see Fig. 1b for quantifications). **c**, Lineage tracing of TGFβ-signalling tumour cells marked at the papilloma stage, traced to the SCC and analysed by FACS shows that TGFβ-responding papilloma cells contribute significantly to SCC tumour progression. **d**, Transcript levels of *Itga6*, *Itgb1* and *Cd44* are increased from normal skin to papilloma and SCC. **e**, UMAP of the number of genes per cell. **f**, Violin plots showing that the quality of samples (with FACS labelled cell identities) in the scRNAseq was high as judged by the number of counts per cell, the number of genes per cell, and the low percentage of the mitochondrial genome captured. **g**, UMAPs of control genes for basal SCC cells (*Itga6, Cd44*), suprabasal tumour cells (*Krt6b*) and SCC-CSCs (*Cd80*) (see Fig. 1c for additional details). All statistics were using unpaired two-tailed Student’s *t*-test: ns, *p* ≥ 0.05); *, *p* ≤ 0.05); **, *p* ≤ 0.01; ***, *p* ≤ 0.001; ****, *p* ≤ 0.0001. Data are presented as mean ± s.e.m. The diagram in **c** was created using BioRender. Source data

To control tumorigenesis, we took embryonic day 9.5 (E9.5) FVB mouse embryos with a tetracycline-inducible RAS oncogene (*TRE-HRAS*^*G12V*^) and performed low-titre in utero lentiviral delivery to selectively transduce a small number of skin basal progenitors with an rtTA3 transactivator and TGFβ reporter under the control of pSMAD2/3–SMAD4-complex-binding elements (SBE) (Fig. 1a). Postnatal doxycycline resulted in clonally transduced skin patches of activated HRAS(G12V). By around 4 weeks, hyperplastic, well-differentiated benign papillomas with smooth undulating epithelial–mesenchymal borders had formed, of which most advanced to undifferentiated, uniformly invasive SCCs by about 8 weeks (Fig. 1a). SCCs displayed only sparse differentiated keratin pearls, while most epithelial–stromal borders were poorly defined. As judged by immunofluorescence imaging and fluorescence-activated cell sorting (FACS), TGFβ signalling and the phosphorylation of its downstream target transcriptional cofactor SMAD2 (pSMAD2) were rare in papillomas but increased substantially in invasive SCC progenitors (Fig. 1b and Extended Data Fig. 1a,b). Taken together with our previous analysis of mixed papilloma–SCC tumours^6^, this result provided an important temporal layer by linking TGFβ signalling to the progression of CSCs from benign to malignant states.

Investigating deeper into the physiological relevance of this temporal change, we added a *creER*^*T2*^ transgene under the control of the SBE-driven reporter and, on the basis of tamoxifen-activated lineage-tracing, we found that, even though the TGFβ-reporter-positive cells were infrequent in papillomas, they contributed substantially to SCCs (Extended Data Fig. 1c). To further dissect the differences, we performed scRNA-seq Smart-seq2 analysis of histologically prevalidated, uniformly invasive SCCs of which the progenitors had been enriched by FACS (Extended Data Fig. 1d and Supplementary Fig. 3). Quality controls revealed sufficient transcriptome detection rates, with ~7,500 genes per cell and low mitochondrial gene contamination (Extended Data Fig. 1e,f).

Transcriptomes fell into three clusters: C1, *Itga6*^low^*Itgb1*^low^*Cd44*^+^ suprabasal cells that had been added as a reference and displayed SCC differentiation markers such as *Krt6b*; and C2 and C3 basal cells, both of which were *Itga6*^high^*Itgb1*^high^*Cd44*^+^ and were expressed at higher levels than normal skin stem cells (Fig. 1c and Extended Data Fig. 1g). Despite morphological uniformity, transcriptional heterogeneity emerged within the basal population of advanced SCCs that had not been previously recognized. This was exemplified by TGFβ-reporter-positive (nuclear mCherry) progenitors that, although found at invasive fronts of mixed tumours^6^, still showed heterogeneity among invasive SCCs, with 57% of C2 cells positive for *mCherry* transcript compared with 35% of C3 cells (Fig. 1c, dotted box). Thus, although enriched for TGFβ signalling, C2 cells were not defined solely by this marker.

## Shifting stem cell–microenvironment crosstalk

Cluster C2 was enriched for *Cd200*, *Hmga2* and *Pthlh*, which were previously shown to typify SCC progenitors enriched for tumour-initiating CSCs^6^. However, this cluster also displayed many other transcripts that are not clearly aligned with previous SCC-CSC signatures (Fig. 1c and Extended Data Fig. 1g). Of 1,894 transcripts enriched in basal SCC cells relative to differentiated tumour cells, 732 were specific to C2 (Supplementary Table 1).

**Extended Data Fig. 2:**
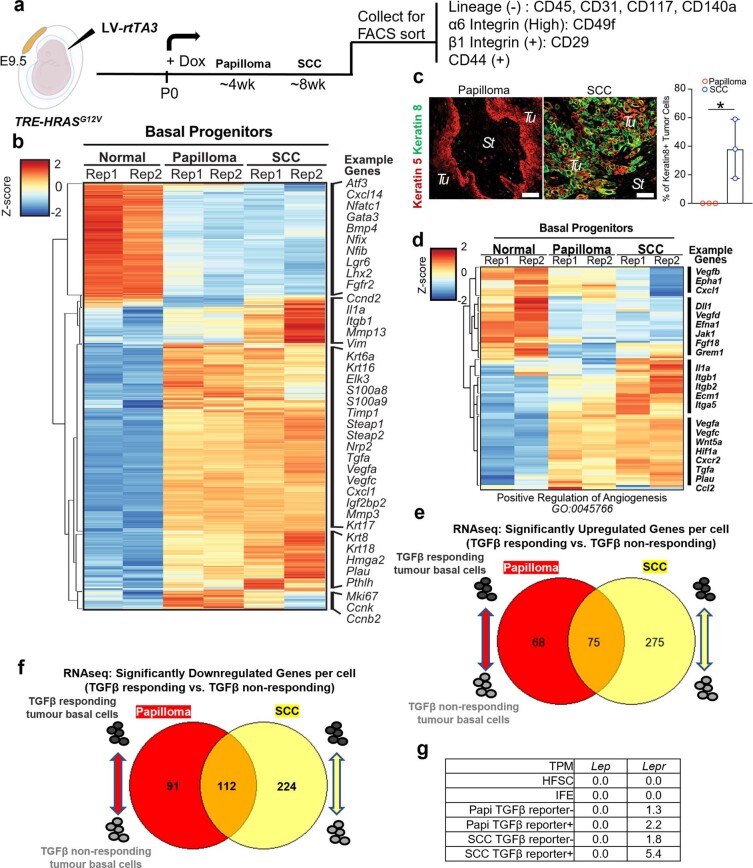
FACS-isolation and transcriptomic analysis of basal progenitors from normal skin, benign papilloma and SCC. **a**, Experimental design for the tumour model as in Fig. 1a, used to purify basal progenitors from papillomas and SCCs. Basal cells (n = 2 mice per condition) are isolated by FACS using tumour basal cell markers (ITGA6, ITGB1, and CD44) with non-epithelial cell types (CD31, endothelial cells; CD45 pan-immune cells; CD117, melanocytes; CD140a, mesenchymal cells) excluded. **b**, Heatmap representation of bulk RNAseq of FACS-isolated basal progenitors from normal skin epithelia, papilloma, and SCC (in replicate) show significant molecular changes and stage-specific signatures during tumour progression. **c**, Immunofluorescence images show that keratin 8 positive tumour cells, as a proxy for the C2 SCC cancer-stem cell signature, are rare in the papilloma stage and much enriched in the invasive SCC stage. (*n* = 3 tumours per stage, *p* = 0.0334). *Tu*, tumour; *St*, stroma. Scale bars,50 µm. **d**, Heat map of the angiogenesis GO-Term. Note: RNAseq in Extended Data Fig. 2b and d, Rep1 SCC displayed mixed SCC-papilloma features. **e**, For high throughput RNA sequencing, two independent replicates of four FACS isolated populations were used. Venn diagram shows the differential expression of genes (DEG) analysis of RNAseq data from TGFβ responding tumour basal cells over their non-responding neighbours and compared between papilloma and SCC. DEG analysis yielded 68 TGFβ responding upregulated genes unique to the papilloma stage, 275 TGFβ responding upregulated genes unique to the SCC stage, and 75 upregulated genes shared by both stages. **f**, DEG analysis yielded 91 TGFβ responding downregulated genes unique to the papilloma stage, 224 TGFβ responding downregulated genes unique to the SCC stage, and 112 downregulated genes shared by both stages. **g**, Transcript levels of *Lep* are below the limits of detection in papillomas and SCCs. And *Lepr* are also not expressed in normal skin SCs. All statistics were using unpaired two-tailed Student’s *t*-test: ns, *p* ≥ 0.05; *, *p* ≤ 0.05); **, *p* ≤ 0.01; ***, *p* ≤ 0.001; ****, *p *≤ 0.0001. Data are presented as mean ± s.e.m. Source data

To place our CSC signature in the context of tumour progression, we performed bulk RNA-seq analysis of FACS-purified basal progenitors from normal skin, papillomas and SCCs, each staged temporally and histologically before processing (Extended Data Fig. 2a,b and Supplementary Fig. 4). Relative to their normal skin counterparts, pan-tumour basal cells upregulated 886 transcripts by at least twofold (adjusted *P* ≤ 0.05; Supplementary Table 2), whereas 562 transcripts were upregulated specifically during the transition from benign to malignant states (Supplementary Table 3). Although a number of C2 transcripts were found in papillomas, many were induced in SCCs, as exemplified by *Krt8* and *Krt18* transcripts and substantiated by immunofluorescence analysis (Extended Data Fig. 2b,c).

**Extended Data Fig. 3:**
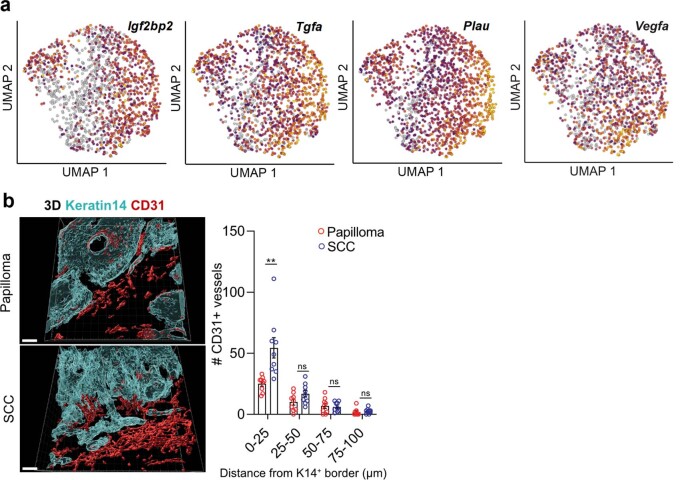
Increased angiogenesis during progression from papilloma to SCC. **a**, UMAP of the examples of angiogenesis-associated mRNAs from scRNA analyses of SCCs that show enrichment in the C2 signature (see Fig. 1c for annotation of clusters). **b**, Collapsed z-stack rendering of clearing and whole-mount immunofluorescence of tissue sections (*n *> 8 tumours per stage). Keratin 14 labels the tumour epithelium; CD31 labels the vasculature. Quantifications are at right. Note that the blood vessel proximity is closer in invasive SCC than papilloma. Scale bars, 40 µm. (*n* = 8 tumours per condition per stage, *p*_(0–25)_ = 0.0034, *p*_(25–50)_ = 0.0801, *p*_(50–75)_ = 0.7548, *p*_(75–100)_ = 0.4734). All statistics were using unpaired two-tailed Student’s *t* test: ns, *p* ≥ 0.05); *, *p* ≤ 0.05); **, *p* ≤ 0.01; ***, *p *≤ 0.001; ****, *p* ≤ 0.0001. Data are presented as mean ± s.e.m. Source data

Further insights into the unique features of tumour-initiating CSCs were revealed by the Gene Ontology (GO) terms of the C2 cluster. Angiogenesis appeared at the top of this list, along with cell migration, wound healing, protein phosphorylation and intracellular signalling (Fig. 1d). Uniform manifold approximation and projection (UMAP) plots highlighted the enrichment of angiogenesis genes in this cluster, many of which were upregulated during the benign–malignant transition (Extended Data Fig. 2d). Consistent with the preponderance of secreted angiogenic factors, reconstructed 3D immunofluorescence images revealed a significant influx in CD31^+^ vasculature specifically at the benign-to-invasive SCC transition (Extended Data Fig. 3a,b). This correlation between C2 cells, invasive SCC fronts and enrichment in angiogenesis was further validated by co-immunolabelling for C2 marker keratin 18 and quantification of CD31^+^ vascular cells (Fig. 1e).

Overall, whereas previous studies reported an increase in vasculature during the transition from normal skin stem cells to papillomas^24^, here we found a notable further increase in the vasculature specifically during the progression to SCCs. This elevation appeared concomitantly with SCC-CSCs and TGFβ signalling, suggesting that these features were functionally intertwined. Further support came from RNA-seq and differential gene expression analysis of FACS-purified papilloma versus SCC progenitors fractionated according to TGFβ reporter activity. Despite their temporal lineage relationship, TGFβ-responding basal SCC cells differed from those of papillomas (Extended Data Fig. 2e,f). These data suggest that progenitors that progress to SCC are influenced by shifting crosstalk with their tumour microenvironment.

**Fig. 2:**
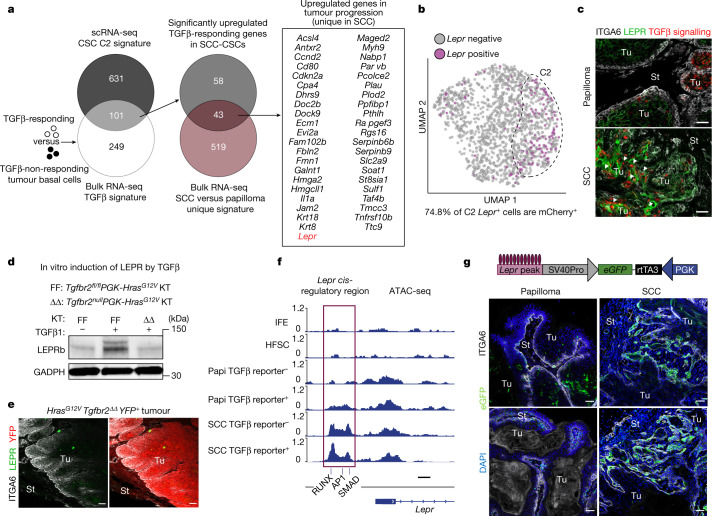
Leptin receptor is a TGFβ-regulated gene induced in tumour-initiating CSCs and localized to invasive SCC fronts. **a**, Venn diagram showing that 101 genes constitute a refined CSC signature shared by single-cell C2 and TGFβ-responsive transcriptomes in SCC basal progenitors (Extended Data Fig. 2). Of the 101 genes, the 43 listed overlap and are upregulated in the papilloma-to-SCC transition. **b**, *Lepr*-expressing cells reside within the C2 basal SCC population and overlap around 75% with TGFβ-reporter^+^ cells. **c**, Immunofluorescence analysis of primary mouse skin SCC confirms that LEPR is rarely expressed in papillomas but is enriched in TGFβ-reporter^+^ SCC cells (arrowheads). Scale bars, 50 µm. **d**, LEPR immunoblot analysis. Cultured *Hras*^*G12V*^ keratinocytes (KT) that are wild type (FF) but not mutant (ΔΔ) for the TGFβ receptor gene (*Tgfbr2*) elevate LEPR substantially in response to active recombinant TGFβ1. GAPDH was used as the loading control. Gel source data are provided in Supplementary Fig. 1a. **e**, Immunofluorescence analysis of tumour tissue from *FR-LSL-Hras*^*G12V*^*;Tgfbr2*^*fl/fl*^*;R26-LSL-YFP* mice transduced at a low titre with *PGK-creER*^*T2*^ lentivirus, and treated with tamoxifen to induce YFP(pseudoRed)^*+*^ *Hras*^*G12V*^*Tgfbr2*^*ΔΔ*^ tumorigenesis. The loss of TGFβ signalling results in non-invasive tumours that do not express LEPR. Scale bars, 50 µm. **f**, ATAC-seq was performed on FACS-purified ITGA6^high^ITGB1^high^ basal populations of interfollicular epidermis (IFE, SCA1^+^), bulge hair follicle stem cells (HFSCs, CD34^+^) and tumour cells (CD44^hi^^gh^) either positive or negative for TGFβ responsiveness (mCherry). ATAC peaks associated with the *Lepr* locus opened during tumorigenesis, with the encased cluster 6 peak (containing RUNX, AP1 and SMAD motifs) opening predominantly during SCC progression. Scale bar, 500 bp. Papi, papilloma. See also Extended Data Figs. 4 and 5. **g**, Schematic of the in vivo *Lepr* ATAC-peak eGFP reporter assay. Reporter activity is greatly enriched at the benign-to-invasive SCC transition. Scale bars, 50 µm. See also Supplementary Tables 4 and 5.

When C2 cells were specifically scored for elevated TGFβ signalling, 101 associated transcripts were also upregulated (Fig. 2a and Supplementary Table 4). In addition to *Cd80*—a factor in resisting immunotherapy^5^—this shortlist included *Ccnd1* and *Ccnd2*, *Hmga2*, *Pcolce2*, *Rgs16*, *St8sia1*, *Tnfaip2* and *Pthlh*, which are known to correlate with stem cell self-renewal/survival, proliferation and/or poor prognosis in SCCs. The list also included *Krt8*, *Krt18*, *Mmp14* and *Mmp1a*, which are implicated in basement membrane remodelling, cytoskeletal dynamics and/or migration/metastasis. Genes encoding angiogenic factors also remained on this list, consistent with active TGFβ emanating from perivascular immune and other stromal cells near invasive fronts^6,19^.

Notably, 43 out of these 101 genes in the TGFβ-signalling SCC-CSC signature were specifically induced/elevated during the transition to SCC (Fig. 2a and Supplementary Table 5). As TGFβ-signalling papilloma progenitors lineage traced to SCC-CSCs, these data implied that CSC gene expression is affected by changes in the tumour microenvironment.

## *Lepr* is an unexpected component of the CSC signature

In considering CSC signature proteins that might be able to sense, respond to and take advantage of the notable changes in the tumour microenvironment at the benign–malignant transition, *Lepr* caught our attention. Traditionally studied in the context of energy balance, LEPR signalling is triggered by its ligand leptin, which is primarily produced by white adipose tissue, but can enter the circulation to reach distal LEPR^+^ target tissues, such as the hypothalamus^25^.

**Extended Data Fig. 4:**
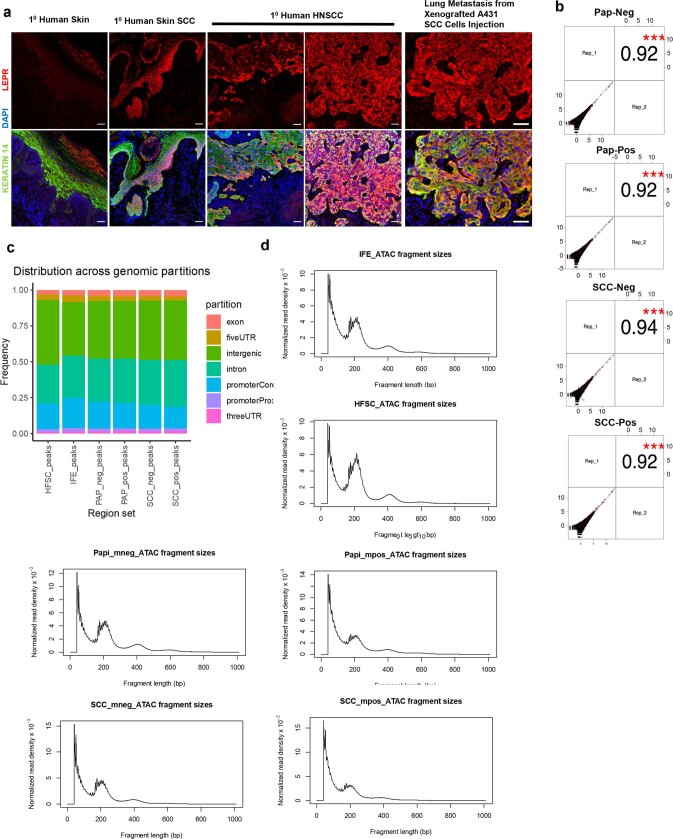
LEPR expression in skin and SCCs from human and quality control for ATAC Sequencing. **a**, LEPR immunofluorescence of human normal skin, head and neck SCC (HNSCC), and lung metastases from human SCC A431 epidermal cells following tail-vein injections in immunocompromised mice. Top row: LEPR labelling alone; bottom row: LEPR, Keratin 14 and DAPI. Scale bars,50 µm. **b**, Correlation plot between ATAC replicates of TGFβ-responding and non-responding SCC and papilloma. All replicates have a correlation coefficiency > 0.92 and *p* < 0.001 (denoted as ***). The test statistic is based on Pearson’s product moment correlation coefficient and follows a *t* distribution with n-2 degrees of freedom. **c**, ATAC peak distribution of all 6 samples according to gene features. All samples display comparable distributions. **d**, Distribution of tagmented fragments in all ATAC-seq samples. Nucleosome laddering is clear in all samples. All statistics were using unpaired two-tailed Student’s *t*-test: ns, *p* ≥ 0.05); *, *p* ≤ 0.05); **, *p* ≤ 0.01; ***, *p* ≤ 0.001; ****, *p* ≤ 0.0001. Data are presented as mean ± s.e.m.

*Lepr* was not expressed in homeostatic skin epithelium and was expressed only rarely in papilloma. Within SCC progenitors, *Lepr* was specifically transcribed in mCherry^+^ TGFβ-signalling C2 CSCs (Fig. 2a,b and Extended Data Fig. 2g). LEPR immunofluorescence corroborated its location in invasive mouse SCCs, and was also found human SCC tumours and xenografts (Fig. 2c and Extended Data Fig. 4a).

To understand the specificity of *Lepr* to C2 TGFβ-signalling cells, we first exposed cultured isogenic TGFβ receptor floxed and null *Hras*^*G12V*^ keratinocytes to recombinant TGFβ1 or vehicle control. Immunoblot analysis underscored the sensitivity of LEPR to TGFβ signalling (Fig. 2d). We further documented this dependency by transducing *FR-LSL-Hras*^*G12V*^;*Tgfbr2*^*fl/fl*^*;R26-LSL-YFP* mice with a *PGK-creER*^*T2*^ lentivirus, and then administering tamoxifen to simultaneously ablate the TGFβ receptor, induce tumorigenesis and activate lineage tracing. Without TGFβ receptor signalling, which is known to be essential for EMT-mediated invasion^6^, only a few rare LEPR^+^ cells were detected by immunofluorescence (Fig. 2e).

**Extended Data Fig. 5:**
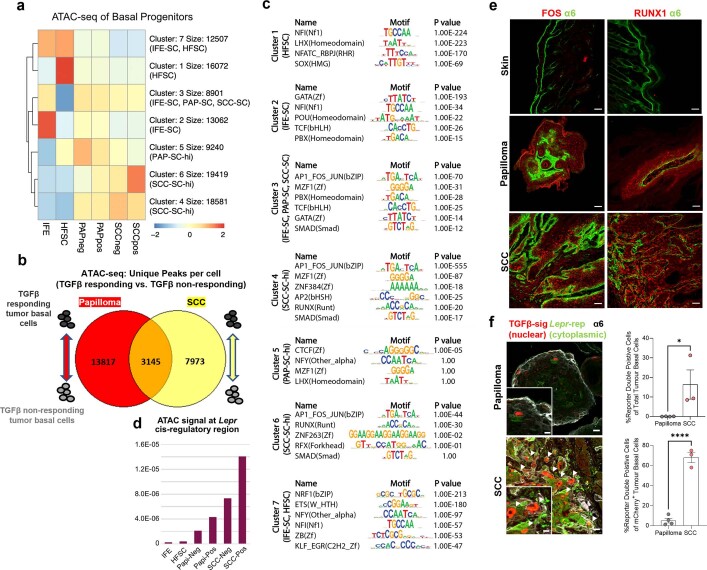
Benign papilloma and invasive SCC display distinct epigenetic signatures. **a**, ATAC sequencing is performed on FACS-purified α6^hi^β1^hi^ basal populations of interfollicular epidermis (IFE, Sca1+), bulge hair follicle stem cells (HFSCs, CD34^+^), and tumour cells (CD44^hi^) either positive or negative for TGFβ-responsiveness (mCherry). Peaks are clustered according to their openness in each population by *k*-mean clustering. **b**, Venn diagram showing marked divergence of ATAC peaks from TGFβ-responding tumour basal cells and their non-responding neighbours between papilloma and SCC stages (*n* = 2 for each condition, each stage). **c**, Motif enrichment analysis of the 7 ATAC peak clusters. **d**, Quantifications of the *Lepr* cis-regulatory region boxed in Fig. 2f. **e**, Immunofluorescence images reveal that transcription factors RUNX1 and FOS are not detected in normal homeostatic skin but are enriched progressively during tumorigenesis. Scale bars, 50 µm. See also pSMAD2 immunofluorescence quantifications in Extended Data Fig. 1a. **f**, *Lepr* EGFP reporter and TGFβ mCherry reporter show minimal activity in papillomas but co-localize at the invasive fronts of SCC. Note numerous SCC cells marked by EGFP cytoplasm and mCherry nucleus. Integrin (white) denotes invasive fronts. For the original images, scale bars, 20 μm. For the magnified insets, scale bar, 10 μm. The percentages of reporter double-positive (DP) cells in these invasive regions are significantly higher in SCC than in papilloma. Majority of the TGFβ mCherry reporter^+^ cells are these DP cells in SCC compared to the ones in papilloma. (*n* = 4 for papilloma, *n* = 3 for SCC; top right: *p* = 0.0477; bottom right: *p* < 0.0001). All statistics were using unpaired two-tailed Student’s *t* -test: ns, *p* ≥ 0.05); *, *p* ≤ 0.05); **, *p* ≤ 0.01; ***, *p* ≤ 0.001; ****, *p* ≤ 0.0001. Data are presented as mean ± s.e.m. Source data

To address whether *Lepr* is a direct transcriptional target of TGFβ receptor signalling in vivo, we performed an assay for transposase-accessible chromatin with high-throughput sequencing (ATAC-seq) analysis of FACS-purified TGFβ-reporter positive versus negative basal tumour populations (Extended Data Fig. 4b–d). Unsupervised clustering of ATAC profiles from purified progenitors of normal skin (interfollicular epidermis; hair follicle), papilloma and SCC revealed seven clusters (Extended Data Fig. 5a).

Peaks in the proximity of *Lepr* mostly fell into clusters 4 and 6, of which the chromatin state displayed marked opening during tumorigenesis, particularly in association with TGFβ-signalling CSCs (Fig. 2f and Extended Data Fig. 5b,c). Within these two peak clusters, AP1 (FOS–JUN) and RUNX1 motifs were enriched, along with canonical pSMAD2/3-binding motifs (21% of C4; 17% of C6). Notably, *Lepr* was among the genes bearing such ATAC peaks and of which the accessibility was sensitive to TGFβ signalling and malignant progression (Fig. 2f and Extended Data Fig. 5d).

Notably, RUNX1 has been shown to be critical for tumour initiation^26^, whereas elevated AP1 (FOS) has been shown to drive basal cell carcinoma to more aggressive SCCs^27^. Similar to pSMAD2, both RUNX1 and FOS showed marked nuclear localization in SCC basal cells at invasive fronts (Extended Data Figs. 1a,b and 5e). pSMAD2/3, the essential co-partner of active TGFβ signalling, best distinguished invasive SCCs from papillomas, suggesting that RUNX1 and AP1 may prime these chromatin peaks while TGFβ signalling drives their activation.

To directly test whether tumour-stage-specific changes in TGFβ signalling govern the chromatin accessibility and expression of *Lepr*, we examined the ability of the C6 *cis*-regulatory element (Fig. 2f, magenta box) to drive temporal activation of an eGFP reporter during tumorigenesis. Interestingly, the *Lepr* reporter was highly active at invasive SCC fronts where TGFβ signalling is high^6^, while much lower in papillomas (Fig. 2g). Consistent with this correlation, in utero co-injection of a TGFβ-signalling *mCherry*^*nuclear*^ reporter and a *Lepr*-*eGFP*^*cytoplasmic*^ reporter revealed that the highest double-fluorescence positivity was among invasive SCC, and the majority of total TGFβ-signalling cells in these regions were positive for the *Lepr-eGFP*^*cytoplasmic*^ reporter in SCC in contrast to papilloma (Extended Data Fig. 5f). These data further underscore the physiological relevance of TGFβ signalling in fuelling the epigenetic dynamics that lead to *Lepr* promoter activation during the transition from the benign to malignant states.

## LEPR functions in malignant progression

**Fig. 3:**
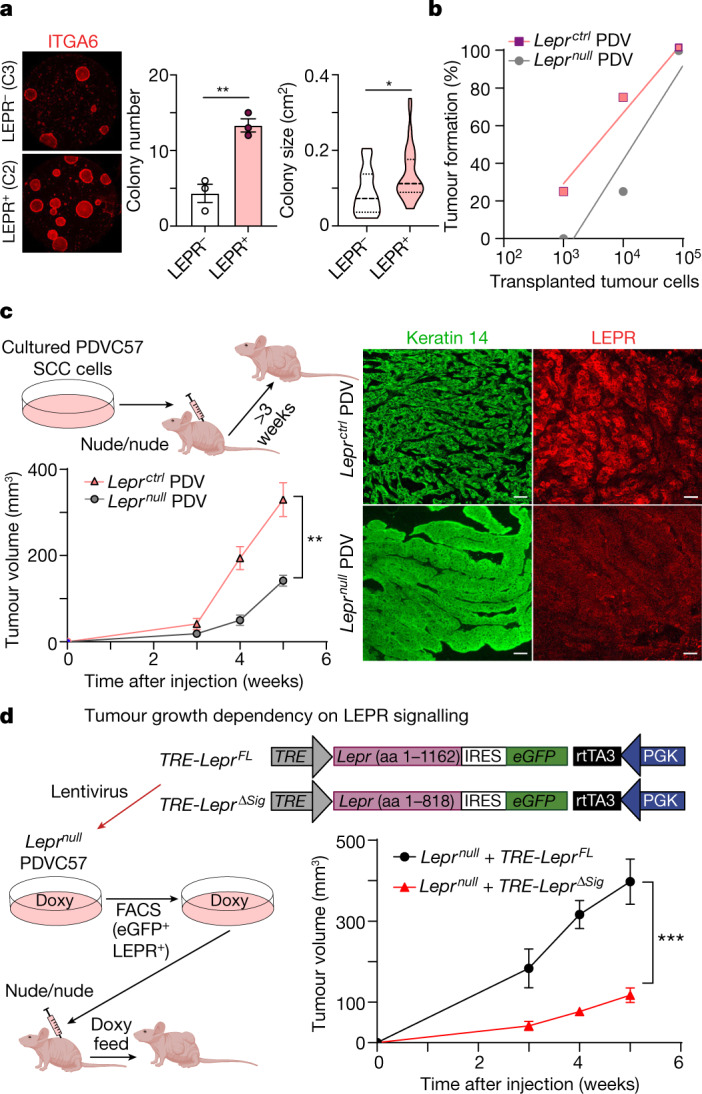
Leptin receptor promotes superior tumour-initiating ability and is an essential regulator of SCC progression. **a**, Stem cell colony assay. When placed in culture, FACS-isolated, LEPR*-*expressing basal SCC progenitors exhibit higher colony-forming efficiency (*n* = 3, *P* = 0.0069) and form larger colonies (*n* = 13 (LEPR^−^), *n* = 39 (LEPR^+^), *P* = 0.0106) compared with non-expressing counterparts. Dish diameter, 10 cm. **b**, Limiting dilution assay. *Lepr*^*null*^ PDVC57 (PDV) SCC cells were generated by CRISPR–Cas9 gene editing (Extended Data Fig. 6b). Serial orthotopic transplantation assays reveal that *Lepr*^*ctrl*^ SCC cells possess higher tumour-initiating ability compared with *Lepr*^*null*^ SCC cells. *n* = 4 (10^5^ and 10^4^ cells) and *n* = 8 (10^3^ cells). **c**, Leptin receptor deficiency impairs SCC progression. Allografted PDV SCC cells were injected intradermally into immunocompromised Nude mice. *Lepr*^*null*^ PDV tumours display reduced growth compared with their control counterparts (*n* = 4, *P* = 0.0039 for the end timepoint). Immunofluorescence shows papilloma-like morphology in *Lepr*^*null*^ PDV tumours and SCC morphology in *Lepr*^*ctrl*^ PDV tumours. Scale bars, 50 µm. **d**, LEPR signalling functions in SCC progression. Lentiviruses containing doxycycline (doxy)-inducible versions of either full-length (FL) *Lepr* or *Lepr*^*Δsig*^ were transduced into *Lepr*^*null*^ PDV SCC cells expressing rtTA3 (required for doxycycline-induced activation of the TRE) (Extended Data Fig. 6d). *Lepr*^*null*^ PDV tumour growth is robust only when full-length *Lepr* but not *Lepr*^*Δsig*^ is reintroduced into tumour cells (*n* = 6, *P* = 0.0008 for the end timepoint), underscoring the need for active LEPR signalling, and not merely LEPR, in tumour growth. For **a**, **c** and **d**, statistical analysis was performed using unpaired two-tailed Student’s *t*-tests. For **a**, **c** and **d**, data are mean ± s.e.m. aa, amino acids. The diagrams in **c** and **d** were created using BioRender. Source data

**Extended Data Fig. 6:**
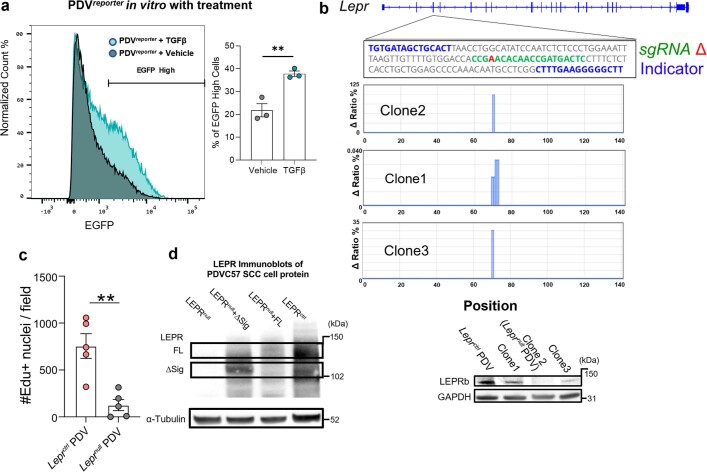
*Lepr* ATAC peak activity is sensitive to TGFβ and to *Lepr* knockout or overexpression. **a**, *Lepr* *cis*-regulatory region reporter (see Fig. 2g and Extended Data Fig. 5f) was transduced into PDV SCC cells and tested for its sensitivity to TGFβ in vitro. Flow cytometry quantifications show that *Lepr* reporter-fluorescence is strongly accentuated in the presence of active recombinant TGFβ1 (*n* = 3, *p* = 0.0068). **b**, *Lepr*^null^ PDVC57 SCC cells were generated by targeted CRISPR/CAS9 technology and validated by iSeq. Blue denotes sequence comparison region; green sgRNA; red, *Lepr* frameshift mutation in Clone 2. MiSeq analysis of *Lepr* targeted Clone 1 (which did not alter LEPR expression), and Clone 3 (which did reduce LEPR expression but not to the extent of Clone 2). Immunoblot (right) shows complete loss of LEPR protein in this clone, which was selected for further study. GAPDH is used as loading control. For gel source data, see Supplementary Fig. 2a. **c**, Quantifications showing reduced proliferation in *Lepr*^null^ compared to *Lepr*^*Ctrl*^ PDV tumours, as judged by EdU-labelling 2 h prior to harvesting. (*n* = 5 tumours per condition, *p* = 0.0024). **d**, Transduced cells are validated by pan-LEPR immunoblot analysis. Brackets denote expected sizes of full-length (FL) LEPR and Δsig LEPR, which lacks the LEPR-signalling domain. α-Tubulin is used as loading control. For gel source data, see Supplementary Fig. 2b. All statistics were using unpaired two-tailed Student’s *t*-test: ns, *p* ≥ 0.05); *, *p* ≤ 0.05); **, *p* ≤ 0.01; ***, *p* ≤ 0.001; ****, *p* ≤ 0.0001. Data are presented as mean ± s.e.m. Source data

Given the association between *Lepr* and C2 SCC-CSCs, we next performed colony-forming assays to test for stemness and found that LEPR^+^ C2 cells showed nearly a threefold higher colony-forming efficiency and formed larger colonies compared with LEPR^−^ C3 cells (Fig. 3a). To functionally test LEPR’s tumour-initiating ability in vivo, we turned to a highly aggressive mouse SCC cell line containing mutations in *Hras* and *Trp53*^28^ (hereafter referred to as PDV). After verifying the TGFβ sensitivity with the *Lepr* reporter in these cells, we used CRISPR–Cas9 editing to generate a *Lepr*-null mutation (Extended Data Fig. 6a,b). Serial-dilution orthotopic transplantation assays on *Lepr*^*null*^ and *Lepr*^*ctrl*^ PDV cells intradermally injected into immunocompromised Nude mice revealed an approximately 10× higher tumour-initiating ability if LEPR was intact (Fig. 3b). Overall, these results suggested that LEPR identifies a subpopulation of TGFβ-signalling, oncogenic-RAS-driven SCC progenitors endowed with heightened stemness and tumour-initiating ability.

Intradermal grafting of our PDV lines in Nude mice revealed *Lepr*^*ctrl*^ tumours displaying features of SCCs by 3 weeks and, by 5 weeks, they reached the maximum allowable size (AALAC regulations). By contrast, *Lepr*^*null*^ PDV tumours were considerably smaller and exhibited papilloma-like morphology (Fig. 3c). As judged by labelling of S-phase cells with thymidine analogue 5-ethynyl-2′-deoxyuridine (EdU), *Lepr* loss reduced, although did not abrogate, proliferation within the tumour (Extended Data Fig. 6c).

## LEPR signalling mediates malignancy

To test whether active LEPR-signalling is required to drive SCC progression, we asked whether we could rescue the inhibitory effects of *Lepr* ablation with an inducible *Lepr* transgene that lacked the encoded cytoplasmic signalling domain of LEPR (ΔSig). Allografts on non-obese host mice revealed that even though transduced full-length LEPR was expressed at lower levels than the control, it restored aggressive SCC tumour growth to PDV *Lepr*^*null*^ cells. By contrast, the expression of LEPR(ΔSig) had little if any effect (Fig. 3d and Extended Data Fig. 6d). Thus, LEPR signalling, and not merely the presence of LEPR, was critical in driving SCC progression of RAS-driven oncogenic stem cells.

## Angiogenesis increases tumour leptin

**Fig. 4:**
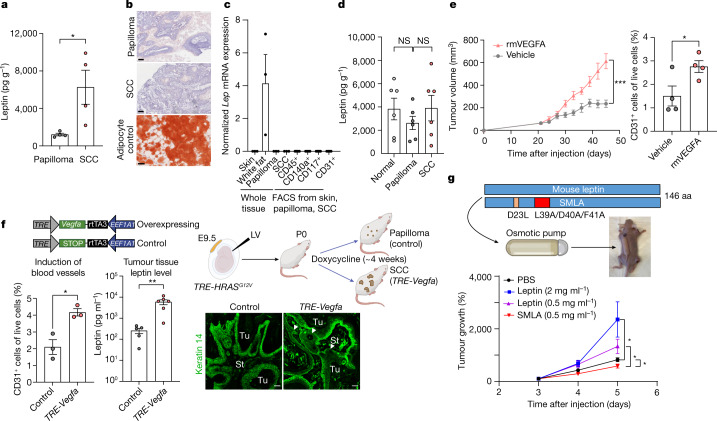
Leptin levels increase in the malignant tumour microenvironment and are caused by elevated angiogenesis. **a**, ELISAs. Leptin in tumour tissue lysates is elevated as papillomas progress to SCC. *n* = 4 tumours per stage. *P* = 0.0322. **b**, Oil red O staining shows no overt signs of mature adipocytes (red) within the stroma surrounding SCCs versus papillomas. Scale bars, 250 µm. **c**, Quantitative PCR reveals no significant *Lep* transcriptional differences in the tumour microenvironments of SCCs versus papillomas. The positive control is *Lep* mRNA from white adipose tissue beneath the normal trunk skin. *n* = 3 (each whole tissue condition), *n* = 5–9 (each FACS-isolated population). **d**, The levels of blood plasma leptin in normal, papilloma and SCC-bearing mice are appreciable, but do not significantly differ. *n* = 6 for each condition. **e**, Tumour growth and angiogenesis are enhanced by intradermal recombinant mouse VEGFA (rmVEGFA), injected every 3 days into PDV SCC tumours and assayed beginning at day 21 after grafting. VEGFA increases the CD31^+^ tumour vasculature, as judged by flow cytometry. *n* = 8 (left) and *n* = 4 (right) tumours per condition. *P* = 0.0002 for the end timepoint (left); *P* = 0.0440 (right). The vehicle control was PBS without mouse VEGFA. **f**, Elevated expression of SCC stem cell C2 signature gene *Vegfa* is sufficient to enhance local angiogenesis and elevate leptin levels in the tumour microenvironment. *TRE-HRAS*^*G12V*^ mice were transduced in utero with low-titre lentivirus containing *EEF1A1-rtTA3* with *TRE-Vegfa* or *TRE-STOP* (schematic). The quantification shows that, after 4 weeks of doxycycline induction, CD31^+^ vasculature (*n* = 3 tumours per stage, *P* = 0.0133) and tissue leptin levels (*n* = 5, control tissues, *n* = 6, *TRE-Vegfa* tissues; *P* = 0.0093) are increased in tumours with CSCs that express elevated *Vegfa*. On the basis of the immunofluorescence analysis, VEGFA over-expressing tumours advance to invasive (arrowhead) SCCs when the controls are still papillomas. Scale bars, 50 µm. **g**, SCC tumour growth is sensitive to plasma leptin levels. Recombinant leptin or mutant SMLA leptin agonist (doses indicated) was delivered to the circulation by an osmotic pump and the effects on PDV SCC tumour growth were monitored for 5 weeks. *n* = 12 (PBS control), *n* = 8, (each LEP or SMLA condition). From top to bottom, *P* = 0.0121, *P* = 0.0194, *P* = 0.0392. Statistical analysis was performed using unpaired two-tailed Student’s *t*-tests (**a** and **d**–**g**). Data are mean ± s.e.m. (**a** and **c**–**g**). aa, amino acids.The diagrams in **f** and **g** were created using BioRender. Source data

As judged by tumour lysate ELISAs, leptin levels were greater than 5× higher in total tumour tissue of SCC relative to papilloma (Fig. 4a). This rise emanated from the tumour microenvironment, as neither the epithelial papilloma nor SCC cells expressed the ligand (Extended Data Fig. 2g). Turning to the source of elevated leptin, we first considered direct delivery from local fat, but saw no overt signs of increased adipogenesis in the tumour microenvironment as judged by Oil red O staining (Fig. 4b). Analogously, neither stroma nor FACS-purified stromal populations displayed appreciable *Lep* mRNA that might account for the rise in leptin protein within the SCC microenvironment (Fig. 4c).

**Extended Data Fig. 7:**
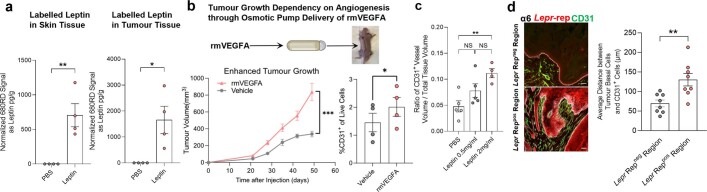
Increased angiogenesis elevates local leptin levels and promotes Lepr-mediated tumour progression. **a**, Fluorescently labelled leptin placed in the circulation can elevate local tissue levels of leptin in the skin and skin tumours. ELISA-like assays show that by delivery of 680RD labelled recombinant leptin through osmotic pump for 1W, fluorescence can be detected in skin and tumour tissue lysatesin a dose-depend manner. (*n* = 4 for each condition; Left: *p* = 0.0055; Right: *p* = 0.0199). **b**, Tumour growth and angiogenesis are enhanced by systemic recombinant mouse mVEGFA, delivered to the circulation by an osmotic pump distant from PDV SCC tumour sites, which are monitored for about 5W. CD31^+^ tumour vasculature is evaluated by flow cytometry (Left: *n* = 8 tumour per condition, *p* = 0.0004 for the end time point; Right: *n* = 4 per condition, *p* = 0.0461, paired due to the nature of angiogeneiss and relative location of pump). Vehicle control is PBS lacking mVEGFA. **c**, Osmotic pump delivery of recombinant protein to elevate circulating leptin levels does not appreciably induce angiogenesis in normal skin. Leptin was administered at two different doses and PBS was used as a control (*n* = 5 for each condition, *p*_(PBS-Lep0.5)_ = 0.1133, *p*_(Lep0.5-Lep2)_ = 0.0865, *p*_(PBS-Lep2)_ = 0.0029). When taken with Fig. 4g, this finding indicates that elevated levels of plasma leptin on its own is sufficient to enhance tumour growth, independent of possible secondary consequences arising from enhanced angiogenesis that might otherwise bring other hormones or growth factors to the surrounding tumour tissue. **d**, Immunofluorescence of tissue sections for *Lepr* reporter and CD31. EGFP labels the CSCs; CD31 labels the vasculature. Quantifications are based on the average distance from the CD31^+^ vasculature to tumour basal cells with or without reporter signalling. (*n* = 8 regions per condition, *p* = 0.0049). All statistics were using unpaired (unless noted) two-tailed Student’s *t*-test: NS, *p* ≥ 0.05); *, *p* ≤ 0.05); **, *p* ≤ 0.01; ***, *p* ≤ 0.001; ****, *p* ≤ 0.0001. Data are presented as mean ± s.e.m. Scale bars, 50 µm. The diagram in **b** was created using BioRender. Source data

As circulating leptin crosses the blood–brain barrier^25^, we next considered the circulation as a possible source of tumour tissue leptin. We first used an osmotic pump to deliver fluorescently labelled leptin to the circulation and verified leptin’s ability to enter both normal skin dermis and tumour stroma from circulation (Extended Data Fig. 7a). Leptin normally circulates through the bloodstream, which we corroborated by enzyme-linked immunosorbent assays (ELISAs) of blood plasma from non-tumour bearing control mice. However, in contrast to obese animals, in which serum leptin is elevated^25,29^, our tumour-bearing mice were not obese, and we did not detect a significant increase in serum leptin during tumour progression (Fig. 4d).

Given these collective results, we examined whether the rise in local vasculature might be at the root of the elevated tissue leptin associated with SCCs. To test this possibility, we first intradermally injected recombinant VEGFA and verified that both angiogenesis and tumour growth were markedly increased (Fig. 4e). To guard against wound-induced effects due to injections, we also validated these effects by osmotic pump implantation to deliver VEGFA systemically (Extended Data Fig. 7b).

As *Vegfa* is an early-activated, C2-enriched CSC gene, we pursued its physiological importance by expressing a doxycycline-inducible *Vegfa* transgene in our *TRE*-*HRAS*^*G12V*^ tumorigenesis model. Notably, *Vegfa* induction in the CSCs directly increased local angiogenesis and invasive tumour behaviour. Most importantly, the ensuing elevated angiogenesis directly elevated leptin levels in the tumour microenvironment (Fig. 4f).

As increasing capillary density might elevate additional hormones and growth factors within the tissue, we used an osmotic pump to directly manipulate leptin levels in the circulation. Using different doses of recombinant leptin as well as a superactive mouse leptin antagonist (SMLA) that abrogates leptin’s signalling activity even when bound to LEPR^30^, we further found that circulating leptin accelerated tumour growth in a dose-dependent manner, whereas SMLA had a slightly repressive effect (Fig. 4g). These findings underscored the ability of circulating leptin on its own to affect tumour progression.

Finally, we did not observe a substantial change in angiogenesis in the skin when we elevated circulating leptin in non-tumour-bearing mice, consistent with the view that, in SCCs, leptin is not the driver but rather the consequence of the elevated angiogenesis that occurs during malignant progression. That said, there was a measurable modest difference, raising the possibility that a feed-forward loop may be operating during malignant progression (Extended Data Fig. 7c). Overall, when coupled with the enhanced proximity of *Lepr* reporter activity to blood vessels in SCC-CSCs (Extended Data Fig. 7d), our results provide compelling support for a model in which increased angiogenesis at the invasive SCC front endows the tumour microenvironment with an ample supply of leptin, while perivascular-associated immune and other stromal cells^6,19^ provide the TGFβ necessary to induce *Lepr* expression in CSCs.

## A LEPR–PI3K–AKT–mTOR path to malignancy

**Fig. 5:**
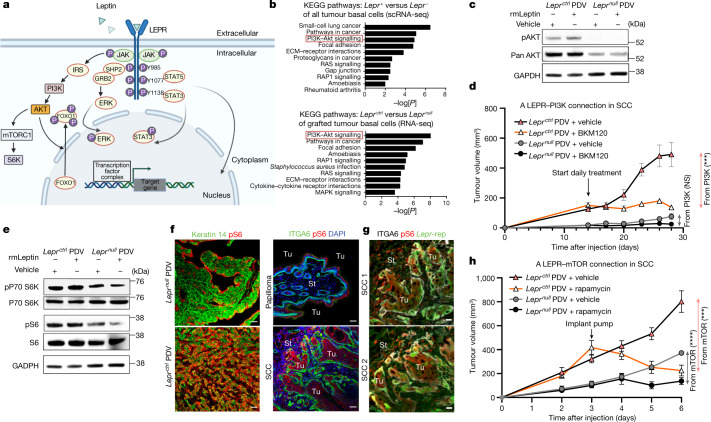
Leptin receptor signalling promotes SCC progression through the PI3K–AKT and mTOR pathways. **a**, Schematic illustrating the complexities of leptin receptor signalling. **b**, The top ten KEGG pathways of genes significantly upregulated in progenitors of *Lepr-*expressing HRAS(G12V) SCCs (data from Fig. 1) (top) and *Lepr*^*ctrl*^ versus *Lepr*^*null*^ PDV tumours (bottom). *P* values were calculated using DAVID bioinformatic analysis. **c**, Immunoblots of protein lysates from *Lepr*^*null*^ and *Lepr*^*ctrl*^ SCC cells treated with recombinant leptin or vehicle control for 48 h before analysis. Note the leptin-dependent activation of pAKT exclusively in LEPR^+^ cells, along with higher AKT levels (Extended Data Fig. 8e). Gel source data are provided in Supplementary Fig. 1b. **d**, Immunocompromised mice with *Lepr*^*ctrl*^ and *Lepr*^*null*^ PDV tumours on opposite sides of their backs were administered the PI3K inhibitor BKM120 or vehicle control daily through oral gavage beginning at 14 days after PDVC57 cell injections. As judged by this assay, most tumour growth attributable to PI3K signalling operates through LEPR. *n* = 6 for each condition. *P* = 0.0576 (*Lepr*^*null*^) and *P* = 0.0007 (*Lepr*^*ctrl*^) at the end timepoint. **e**, Immunoblotting reveals signs of mTORC1 pathway elevation (pS6 and pS6-kinase) after leptin–LEPR signalling in vitro. An identical GAPDH image from Fig. 5c is displayed here as a reference, as they are from the same experiment. Gel source data and experiment details are provided in Supplementary Fig. 1b. **f**, The importance of leptin–LEPR signalling in activating mTORC1 signalling is accentuated in vivo, where the background from other growth factors in enriched medium is eliminated. pS6 immunofluorescence reveals LEPR dependency on mTORC1 activity in PDV-engrafted tumours and particularly pronounced activity at the invading fronts of LEPR^+^ *HRAS*^*G12V*^ SCCs. Scale bars, 50 µm. **g**, pS6 immunofluorescence (mTORC1 activity) and *Lepr* eGFP reporter (rep) activity co-localize in cells at invading *HRAS*^*G12V*^ SCC fronts. Scale bars, 20 µm. **h**, Immunocompromised mice with *Lepr*^*ctrl*^ and *Lepr*^*null*^ PDV tumours on opposite sides of their backs were continuously administered rapamycin or vehicle control at *t* = 3 weeks and then monitored for tumour progression. As judged by this assay, most tumour growth attributable to mTOR signalling operates through LEPR. *n* = 6 (each condition). *P* < 0.0001 (*Lepr*^*null*^) and *P* = 0.0002 (*Lepr*^*ctrl*^) at the end timepoint. For **d** and **h**, statistical analysis was performed using unpaired two-tailed Student’s *t*-tests. Data are mean ± s.e.m. (**d** and **h**). The diagram in **a** was created using BioRender. Source data

In other cellular contexts, LEPR signalling relies on its association with the Janus kinase (JAK2), which, after leptin-LEPR binding, phosphorylates LEPR’s intracellular domain. Once phosphorylated, LEPR has been implicated in activating various downstream pathways, including signal transducer and activator of transcription 3 (STAT3) and PI3K^31,32^ (Fig. 5a).

**Extended Data Fig. 8:**
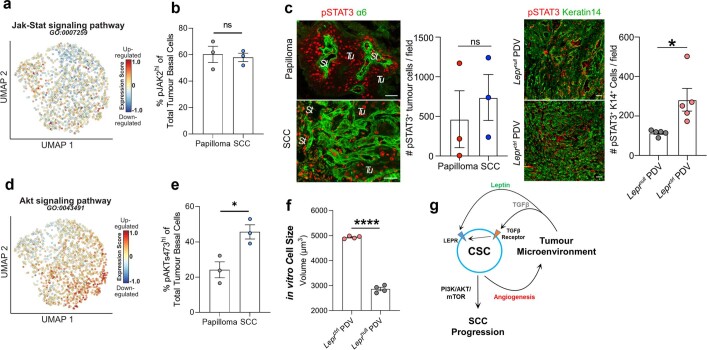
Akt but not Jak/Stat pathway signature is enriched in SCC CSCs. **a**, The Jak-Stat pathway mRNA signature is not enriched in any of the three scRNAseq SCC clusters. **b**, Flow cytometry for pJAK2 reveals that JAK-signalling is activated in skin tumours, but is not significantly changed between papillomas and SCCs. (*n* = 3 for independent tumours per stage. *p* = 0.7565 with two-sided unpaired Student’s *t*-test.). **c**, pSTAT3 immunofluorescence shows that although not detected in homeostatic skin, STAT3 is activated similarly in both papilloma and SCC (left). pSTAT3 is reduced but not abolished in *Lepr*^*null*^ compared to *Lepr*^*Ctrl*^ PDV tumours (right), suggesting that LEPR’s main role in SCC tumour progression is not to activate STAT3. Scale bars, 50 µm. Quantifications accompany each analysis. (Left: *n* = 3 for tumours per stage, *p* = 0.5835; Right: *n* = 5 for tumours per condition, *p* = 0.0195. All are independent samples.). **d**, *Lepr* downstream signalling Akt pathway mRNA signature is enriched in C2 cluster of scRNAseq of SCC. **e**, Flow cytometry reveals that the percentage of pAKTs473 cells is higher in SCC than papilloma. (*n* = 3 for independent tumours per stage, *p* = 0.0237). **f**, *Lepr*^ctrl^ PDV cells are significantly larger in size compared to *Lepr*^null^ PDV cells (*n* = 4 for each condition, *p* < 0.0001). **g**, Schematic summarizing our findings. During tumour progression, dynamic crosstalk between HRAS^G12V^ oncogenic epithelial SCs and their tumour microenvironment promotes an increase in the production of angiogenesis factors by emerging SCC-CSCs, which in turn fuels angiogenesis, elevating the levels of circulating factors, such as leptin by increasing vasculature density. The perivasculature also raises local immune cells that elevate local TGFβ levels. Enhanced TGFβ-signalling in the CSCs not only promotes an EMT-like invasion^6^, but also activates *Lepr* transcription. This triggers a leptin-LEPR signalling cascade, elevating PI3K-AKT-mTORC signalling and fuelling SCC progression. The genes in this cascade are often found mutated in cancers, but as shown here, can be driven by interactions between CSCs and their tumour microenvironment. See also Fig. 5a. All statistics were using unpaired two-tailed Student’s *t*-test: ns, *p* ≥ 0.05); *, *p* ≤ 0.05); **, *p* ≤ 0.01; ***, *p* ≤ 0.001; ****, *p *≤ 0.0001. Data are presented as mean ± s.e.m. Source data

At the transcriptional level, the JAK–STAT signature showed no enrichment in our SCC-CSCs and, although flow cytometry verified JAK2 phosphorylation, the differences between papilloma and SCC, while variable, were not significant (Extended Data Fig. 8a,b). STAT3 was also phosphorylated and present in the nucleus in papillomas and, although pSTAT3 was diminished in *Lepr*^*null*^ PDV tumours, it was not abrogated (Extended Data Fig. 8c). Thus, LEPR–leptin signalling appeared to act as a catalyst to enhance, not induce, JAK–STAT signalling to a level that facilitated progression from the benign to invasive state in SCCs.

Turning to an unbiased approach to delve further into mechanism, we analysed our transcriptomes of individual SCC basal cells according to their level of *Lepr* expression. On the basis of Kyoto Encyclopedia of Genes and Genomes (KEGG) pathway analysis, the top three pathways distinguishing *Lepr*^*+*^ versus *Lepr*^*−*^ basal SCC cells were small-cell lung cancer (oncogenic RAS-associated), pathways in cancer and the PI3K–AKT signalling pathway (Fig. 5b, top). Indeed, comprehensive gene signature expression scores for the AKT signalling pathway showed significant upregulation in C2 SCC-CSCs, with a marked elevation between papilloma and SCC states (Extended Data Fig. 8d,e).

To examine the PI3K–AKT connection further, we performed bulk RNA-seq analysis of FACS-purified basal cells from tumours that developed from our engrafted PDVC57 cells. KEGG analysis placed the PI3K–AKT signalling pathway at the top of molecular features that distinguished *Lepr*^*ctrl*^ versus *Lepr*^*null*^ tumours (Fig. 5b, bottom). Taken together, LEPR-PI3K–AKT surfaced as a top candidate for a signalling pathway that could account for the heterogeneity in our basal progenitor population of invasive SCCs.

In vitro, *Lepr*^*ctrl*^ but not *Lepr*^*null*^ SCC cells were sensitive to AKT–PI3K signalling in the presence of recombinant leptin. As judged by immunoblot analyses, both AKT stability and activation (phosphorylation) were enhanced by leptin, but only if SCC cells expressed LEPR (Fig. 5c). Moreover, when we blocked PI3K signalling directly in vivo, the oral PI3K inhibitor BKM120^33^ reduced tumour growth in only *Lepr*^*ctrl*^ SCC and not in *Lepr*^*null*^ SCC (Fig. 5d). Considering the many routes through which PI3K–AKT can be activated, its robust link to LEPR signalling in driving oncogenic RAS tumours to an invasive SCC state was surprising and suggested that, in this context, LEPR–leptin signalling has a profound role in orchestrating the PI3K–AKT cascade and fuelling SCC tumour growth.

Through mechanisms that vary depending on cellular circumstances, PI3K–AKT signalling can lead to the activation of mTOR—a central metabolic mediator in some cancers^34–36^. In agreement, *Lepr*^*ctrl*^ PDV cells in vitro were larger in size compared with *Lepr*^*null*^ PDV cells (Extended Data Fig. 8f). Moreover, both mTOR target, the serine/threonine kinase p70-S6K, and ribosomal protein S6 (a proxy for active p70-S6K and enhanced protein synthesis at the ribosome)^37^ displayed phosphorylation in a *Lepr*-sensitive manner (Fig. 5e).

The importance of LEPR in regulating PI3K–AKT–mTOR in SCC-CSCs extended to in vivo tumours. Thus, tumours arising from engrafted *Lepr*^*null*^ PDV cells displayed reduced pS6 immunofluorescence compared with aggressive SCCs derived from *Lepr*^*ctrl*^ PDV engraftments (Fig. 5f). Furthermore, in the HRAS(G12V)-mediated transition from papilloma to SCC, pS6 was elevated at invasive SCC fronts and, when imaged with our *Lepr* reporter, pS6 and eGFP showed considerable overlap in these regions (Fig. 5g).

Finally, continuous delivery of the potent mTOR inhibitor rapamycin resulted in reduced growth of tumours derived from engrafted *Lepr*^*ctrl*^ PDV cells (Fig. 5h). By contrast, rapamycin had less effect on *Lepr*^*null*^ tumours, the growth of which was already restricted by LEPR loss of function. Notably, although the GO terms for LEPR sensitivity pointed to the PI3K–AKT pathway, AKT can also be phosphorylated by mTORC1, leaving open the possibility of feedback mechanisms arising downstream of LEPR signalling.

## Discussion

Human studies on SCCs have centred largely around invasive metastatic cancers, which often contain a myriad of oncogenic mutations. However, the tumour microenvironment can be equally impactful in driving malignant progression, as exemplified by the effects of obesity on cancer^14,38,39^. In the attempt to identify obesity-driven tumour susceptibility pathways that might alter energy balance, leptin–LEPR signalling has been a focus of cancers of which the normal stem cells express LEPR and exist in a fatty tissue microenvironment in which local leptin is high^35,40,41^. For cancers such as SCCs that originate from native tissues that do not express LEPR, reports of LEPR expression have relied mostly on immunolabelling with antibodies of unclear specificity^42–44^.

How alterations in LEPR signalling contribute to tumour progression and metastasis has remained unclear. Mechanistic insights have relied on cultured cancer cell lines, in which different possible routes have been proposed^35,43,45^ (Fig. 5a). Moreover, it was recently demonstrated that obesity generated by leptin deficiency in mice can affect KRAS-induced pancreatic cancer progression not through impaired LEPR-signalling but, rather, through an obesity-specific mechanism involving aberrant endocrine–exocrine signalling in the adapting pancreatic beta cells^14^.

In our in vivo studies, we did not use an obesity model, nor did we focus on a naturally adipose-rich tissue microenvironment. Rather, we uncovered a cancer link to the leptin–LEPR signalling pathway that becomes activated de novo downstream of an oncogenic HRAS(G12V)-induced change within otherwise normal skin stem cells. In marked contrast to oncogenic KRAS-induced pancreatic cancers, which are influenced heavily by obesity but not leptin^14^, or to pathogen infections that can elicit transient changes in local adipose tissue/leptin levels that affect wound repair^46^, malignant progression in HRAS-induced cutaneous cancers requires the induction of LEPR signalling by the stem cells, but neither obesity nor adipogenesis in the local tissue environment.

LEPR signalling during SCC progression appears to be rooted in two events: first, a CSC-mediated influx of vasculature within the tumour microenvironment that increases blood vessel density at the invasive front and in turn causes local leptin levels to rise within the tumour stroma; and second, a corresponding rise in perivascular TGFβ that enhances TGFβ signalling and *Lepr* gene expression within neighbouring SCC-CSCs. Thus, through the ability of oncogenic RAS to reroute the stem cell’s communication circuitry with its surrounding microenvironment, and the ability of the microenvironment in turn to induce a membrane receptor on the stem cells, CSCs exploit this dynamic crosstalk, fuelling non-genetic circuitries that drive malignant progression (Extended Data Fig. 8g). How leptin transits across the vasculature remains unclear^25,29^, although it is intriguing to speculate that, for solid tumours such as SCCs, mechanical pressures might alter the vascular integrity and facilitate entry of circulating factors such as leptin into the tumour microenvironment.

In summary, the acquisition of an oncogenic RAS mutation sparks the perfect crosstalk between tumour-initiating cells and their microenvironment, enabling them to hijack the LEPR-signalling pathway and fuel cancer progression. In this regard, the downstream consequences of LEPR signalling, namely sustained activation of the PI3K–AKT–mTOR pathway, become all the more important because, among human cancers, *PI3KCA* is among the most commonly mutated genes and a target of emerging anti-cancer therapeutics^7,36^. Our findings raise the tantalizing possibility that *PIK3CA* mutations may not be essential to sustain the PI3K pathway at a level required for malignancy, even though mutational burden may help to bolster it. Similarly, although polymorphisms in *Lep* and *Lepr* have been associated with oral SCCs^47^, our data clearly show that, even if a causal link emerges in the future, such genetic alterations are not required to initiate signalling. Rather, an oncogenic RAS mutation has the ability to launch an aberrant dialogue between the SCs and their normal tissue microenvironment. Furthermore, as SCC-CSCs emerge, they co-opt many of the same signalling pathways achieved by a high mutational burden—a feature with profound implications for our understanding of cancer.

## Methods

### Animals

*TRE-HRAS*^*G12V*^ mice have been described previously^48^. The original *TRE-HRAS*^*G12V*^ C57Bl/6 mice have been backcrossed 10 generations to an FVB/N background. FVB/N *TRE-HRAS*^*G12V*^ mice were bred to FVB/N *R26-LSL-YFP* mice to create the TGFβ-reporter lineage-tracing model. For the *Tgfbr2*-cKO experiment, *FR-LSL-Hras*^*G12V*^*;Tgfbr2*^*fl/fl*^*;R26-LSL-YFP* mice were crossed in-house. For tumour transplantation experiments, 7–9-week-old female NU/NU Nude mice from Charles River were used. All other studies used a mix of male and female mice, which for the assays used here, behaved similarly. The animals were maintained and bred under specific-pathogen-free conditions at the Comparative Bioscience Center (CBC) at The Rockefeller University, an Association for Assessment and Accreditation of Laboratory Animal Care (AALAC)—an accredited facility. Adult animals were housed in a cage with a maximum of five mice unless specific requirements were needed. The light cycle was from 07:00 to 19:00. The temperature of the animal rooms was 20–26 °C, and the humidity of the animal rooms was 30–70%. All mouse protocols were approved by the Institutional Animal Care and Use Committee (IACUC) at The Rockefeller University.

As tumours began to progress to the malignant stage (size > 10 mm), mice were housed individually and antibiotic cream was applied to the surface of the ulcerated tumour. When tumour sizes approached 15 mm, intraperitoneal injection of Bup was used every 8 h to minimize pain. Mice were euthanized once the tumour size exceeded 20 mm or if mice showed any signs of distress, for example, difficulty in breathing.

### Cell lines

The mouse cutaneous SCC cell line PDVC57 was cultured in the E-low medium (E.F.’s laboratory)^5^. Mouse keratinocyte cell line FF (*Tgfbr2*^*f/f*^*PGK-Hras*^*G12V*^) and ΔΔ (*Tgfbr2*^*null*^*PGK-Hras*^*G12V*^) were cultured with the E-low medium as previously discribed^6^. The HNSCC cell line A431 was cultured in DMEM medium (Gibco) with 10% FCS, 100 U ml^−1^ streptomycin and 100 mg ml^−1^ penicillin. The HEK 293TN cell line for lentiviral production was cultured in DMEM medium supplemented with 10% FCS (Gibco), 1 mM sodium pyruvate, 2 mM glutamine, 100 U ml^−1^ streptomycin and 100 mg ml^−1^ penicillin. The 3T3J2 fibroblast feeder cell line was expanded in DMEM/F12 medium (Thermo Fisher Scientific) with 10% CFS (Gibco), 100 U ml^−1^ streptomycin and 100 mg ml^−1^ penicillin. It was then treated with 10 µg ml^−1^ mitomycin C (Sigma-Aldrich) for 2 h to achieve growth inhibition.

The human skin SCC line A431 was from ATCC; mouse skin SCC PDVC57 was a gift from the original laboratory that created it (Balmain lab); mouse keratinocyte cell lines FF (*Tgfbr2*^*f/f*^*PGK-Hras*^*G12V*^) and ΔΔ (*Tgfbr2*^*null*^ *PGK-Hras*^*G12V*^) were generated in E.F.’s laboratory; mouse fibroblast 3T3/J2 has been passaged in the laboratory as feeder cells and originated from the laboratory of H. Green;  HEK 293TN cells were purchased from SBI directly as low passage (P2) for lentiviral packaging. PDVC57 was validated by karyotyping and grafting tests. Mouse keratinocyte cell lines were validated previously in E.F.’s laboratory. 3T3/J2 has been functionally and morphologically validated as feeder cells. HEK 293TN cells were functionally tested as packaging cells producing lentivirus. A431 was not authenticated.

### Human tumour samples

Human skin and SCC tumour samples were acquired as frozen tissue from B. Singh at Weill Cornell Medical College. All of the samples were de-identified according to National Institutes of Health and Federal/State regulations. Informed consent was obtained from all human research participants at Weill Cornell Medical College, and in accordance with approved Institutional Review Broad (IRB) protocols from The Rockefeller University, Weill Cornell Medical College and Memorial Sloan Kettering Cancer Center.

### Lentiviral in utero transduction

Lentiviral constructs were previously described (*SBE-NLSmCherry-P2A-CreERT2 PGK-rtTA3*)^6^ or cloned in E.F.’s laboratory (*SBE-NLSmCherry PGK-rtTA3*, *Lepr* peak reporter-*eGFP PGK-rtTA3*, *TRE-Lepr-IRES-eGFP*, *PGK-rtTA3*, *TRE-Vegfa* *EEF1A1-rtTA3*, *TRE-STOP EEF1A1-rtTA3*). The lentiviral production and in utero injection were performed as previously described^6,23^. In brief, pregnant female mice with a doxycycline-inducible *HRAS*^*G12V*^ transgene were anaesthetized with isoflurane (Hospira) when their embryos were at E9.5. Lentivirus (500 nl to 1 µl) was injected into the amniotic sacs of the embryos to selectively transduce a small number of individual epidermal progenitors within the surface monolayer that gives rise to the skin epithelium^49^. Postnatal induction of tumorigenesis in clonal patches was achieved by doxycycline administration (2 mg per g) through the feed.

### Tumour formation and grafting

To induce spontaneous tumour formation, transduced *TRE-HRAS*^*G12V*^ or *TRE-HRAS*^*G12V*^ *R26-LSL-YFP* mice were continuously fed doxycycline-containing chow (2 mg per g) from postnatal day 0 to 4 to activate the rtTA3 transcription factor and induce tumorigenesis. Papillomas appeared by around 4 weeks and progressed to SCCs by about 8 weeks. To activate the *creER*^*T2*^ in lineage-tracing experiments, 100 μg tamoxifen (Sigma-Aldrich) was injected intraperitoneally into tumour-bearing mice daily for 3 consecutive days. For tumour allograft studies, 1 × 10^5^ mouse PDVC57 SCC cells were mixed with growth-factor-reduced Matrigel (Corning) and intradermally injected into NU/NU Nude immunocompromised mice. Visible tumours appeared after 3 weeks. For metastatic tumour xenografts, 1 × 10^5^ human SCC A431 cells were resuspended in sterile PBS and tail-vein injected into immunocompromised Nude mice. Mouse lung tissue with metastatic lesions was collected after 3 weeks. The volume of the tumour was calculated using the following formula: $\frac{4}{3}\pi \left(\frac{x}{2}\times \frac{y}{2}\times \frac{z}{2}\right)$, where *x*, *y* and *z* are three-dimensional diameters measured using digital callipers (FST).

### Immunofluorescence and histology

For both histology and immunofluorescence analysis, tumour tissues were fixed in 4% PFA at room temperature for 15 min, and then washed three times with PBS at 4 °C. For histology, samples were dehydrated in 70% ethanol overnight, and were sent to Histowiz for Oil Red O and H&E staining. For immunofluorescence, after PBS washes, the samples were dehydrated in 30% sucrose in PBS solution overnight at 4 °C. The dehydrated tissues were embedded in OCT medium (VWR). Cryosections (10 µm) were blocked in PBS blocking buffer with 0.3% Triton X-100, 2.5% normal donkey serum, 1% BSA, 1% gelatin. After blocking, the sections were stained with primary antibodies: ITGA6 (rat, 1:2,000, BD), RFP/mCherry (guinea pig, 1:5,000, E.F.’s laboratory), K14 (chicken, 1:1,000, BioLegend), CD31 (rat, 1:100, BD Biosciences), K5 (guinea pig, 1:2,000, E.F.’s laboratory), K8 (rabbit, 1:1,000, E.F.’s laboratory), mLEPR (goat, 1:200, R&D Systems), hLEPR (rabbit, 1:100, Sigma-Aldrich), RUNX1 (rabbit, 1:100, Abcam), FOS (rabbit, 1:100, Cell Signalling), GFP (chicken, 1:500, BioLegend), pSTAT3-Y705 (rabbit, 1:100, Cell Signalling), pSMAS2-S465/467 (rabbit, 1:1,000, Cell Signalling) or pS6-S240/244 (rabbit, 1:100, Cell Signalling). For pSTAT3 immunolabelling, sections were pretreated with ice-cold 100% methanol for 30 min before blocking. After primary antibody staining, all sections were washed three times with PBS wash buffer containing 0.1% Triton X-100 for 5 min at room temperature. For pSMAD2 immunolabelling, sections were pretreated with 3% H_2_O_2_ for 1 h before blocking, stained using the appropriate HRP-conjugated secondary antibody (Jackson ImmunoResearch) and amplified using the TSA plus Cy3 kit (Akoya Biosciences) in combination with other regular co-stains. The sections were then labelled with the appropriate Alexa 488-, 546- and 647-conjugated secondary antibodies (Thermo Fisher Scientific) and imaged using the Zeiss Axio Observer Z1 with Apotome 2 microscope. Images were collected and analysed using Zeiss Zen software.

For immunofluorescence microscopy of thick tumour sections, all collected tumours were fixed with 1% (v/v) paraformaldehyde/PBS overnight at 4 °C and washed three times with PBS. After an overnight incubation with 30% (w/v) sucrose/PBS at 4 °C and embedding in OCT, 100 μm cryosections were washed with PBS and transferred to a 24-well dish. After overnight permeabilization with 0.3% Triton X-100/PBS at room temperature with rotation, tissue was blocked for 4–6 h with 5% donkey serum and 1% bovine serum albumin in 0.3% Triton X-100/PBS (blocking buffer). Tissue was then incubated with the following primary antibodies for 2 days at room temperature: mCherry (Abcam, 1:1,000), CD31 (Sigma-Aldrich, 1:300), keratin 14 (E.F.’s laboratory, 1:400), Keratin 18 (rabbit, 1:300, E.F.’s laboratory), GFP (chicken, 1:300, E.F.’s laboratory) and ITGA6 (Rat, 1:300, BD Biosciences) before several washes with 0.3% Triton X-100/PBS. The tissue sections were incubated with secondary antibodies (Alexa Fluor-RRX, -488 or -647 hamster, rat, chicken and rabbit at 1:1,000) diluted in blocking buffer overnight (16–20 h) together with DAPI at room temperature and washed with 0.3% Triton X-100/PBS with several exchanges. Immunolabelled tissue sections were then dehydrated with a graded ethanol series by incubation in 30% ethanol, 50% ethanol and 70% ethanol, each set to pH 9.0 as described previously^50^ for 1 h per solution, before a 2 h incubation with 100% ethanol, and cleared to optimize optical sectioning and imaging penetration by overnight incubation with ethyl cinnamate (Sigma-Aldrich). Cleared tumour samples were imaged in 35 mm glass-bottom dishes (Ibidi) with an inverted LSM Zeiss 780 laser-scanning confocal microscope and/or Andor dragonfly spinning disk. Images were then analysed using Imaris imaging software (Bitplane). The shortest distance and volume measurements were performed by the creation of individual objects of CD31^+^ blood vessels, K14^+^ tumour mass, K18^+^ tumour cells or *Lepr* reporter^+^ tumour cells.

### Cell sorting and flow cytometry

To sort the target tumour cell populations by FACS, tumours were first dissected from the skin and finely minced in 0.25% collagenase (Sigma-Aldrich) in HBSS (Gibco) solution. The tissue pieces were incubated at 37 °C for 20 min with rotation. After a wash with ice-cold PBS, the samples were further digested into a single-cell suspension in 10 ml 0.25% trypsin/EDTA (Gibco) for 10 min at 37 °C. The trypsin was then quenched with 10 ml FACS buffer (5% FCS, 10 mM EDTA, 1 mM HEPES in PBS). The single-cell suspension was centrifuged at 700 rcf. The pellet was resuspended in 20 ml FACS buffer and strained through a 70 μm cell strainer (BD Biosciences). The filtered samples were centrifuged at 700 rcf to pellet cells, and the supernatant was discarded. The cell pellet was then resuspended in primary antibodies. A cocktail of antibodies against surface markers at the predetermined concentrations (CD31–APC, 1:100, BioLegend; CD45–APC, 1:200, BioLegend, CD117–APC, 1:100, BioLegend; CD140a–APC, 1:100, Thermo Fisher Scientific; CD29–APCe780, 1:250, Thermo Fisher Scientific; CD49f–PerCPCy5.5, 1:250, BioLegend; CD44–PECy7, 1:100, BD Biosciences) was prepared in the FACS buffer with 100 ng ml^−1^ DAPI. Furthermore, CD44–BV421 (1:100, BD Biosciences), CD49f–PECy7 (1:250, BioLegend) and CD29–APCCy7 (1:250, BioLegend) were also used as interchangeable staining in the panels for the same purpose. The samples were incubated on ice for 30 min, washed with FACS buffer twice and resuspended in FACS buffer with 100 ng ml^−1^ DAPI before FACS and analysis.

To sort the skin stem cell populations (IFE and HFSCs), whole back skins were first dissected from the mouse. After scraping off the fat tissues from the dermal side, the tissues were incubated in 0.25% trypsin/EDTA (Gibco) for 45–60 min at 37 °C. After quenching the trypsin with cold FACS buffer, the epidermal layer and hair follicles were scraped off the epidermal side of the skin. The tissues were mechanically separated/strained into a single-cell suspension for staining. A cocktail of antibodies for surface markers at the predetermined concentrations (CD31–APC, 1:100, BioLegend; CD45–APC, 1:200, BioLegend; CD117–APC, 1:100, BioLegend; CD140a–APC, 1:100, Thermo Fisher Scientific; CD29–APCe780, 1:250, Thermo Fisher Scientific; CD49f–PerCPCy5.5, 1:250, BioLegend; CD34–BV421, 1:100, BD Biosciences; CD200–PE, 1:100, BioLegend; SCA1–PECy7, 1:100, BioLegend) was prepared in the FACS buffer with 20 ng ml^−1^ DAPI when using an ultraviolet laser. The sorting was performed on BD FACS Aria equipped with FACSDiva software.

For the in vivo *Lepr* reporter SCC cell experiment, reporter PDVC57 cells were treated with TGFβ1 (10 ng ml^−1^) for 7 days. The treated reporter PDVC57 cells were stained with 100 ng ml^−1^ DAPI in FACS buffer and analysed on the BD Biosciences LSR Fortessa system together with the control treatment (BSA only).

For the phosphorylated protein flow cytometry experiment, single-cell suspensions were obtained from papilloma or SCC tumour tissues as described above. After washes with cold PBS, cells were stained with Live/Dead Blue (1:200, Thermo Fisher Scientific) on ice for 30 min, and then washed with FACS buffer and blocked with FACS buffer with 5% normal mouse serum (Thermo Fisher Scientific), 5% normal rat serum (Thermo Fisher Scientific) and 1× Fc Block (BioLegend) for 15 min on ice. The live cells were then stained with FITC-conjugated surface marker (DUMP) antibodies (CD31, CD45, CD117, CD140a) (Thermo Fisher Scientific and BioLegend) for 30 min. After washing with FACS buffer, cells were fixed with 1× Phosflow Lyse/Fix Buffer (BD Biosciences) at 37 °C for 10 min. After centrifuging and another wash with FACS buffer, the fixed cells were permeabilized with −20 °C prechilled Phosflow Perm Buffer III (BD Biosciences) for 30 min on ice. The samples were then washed twice with 1× Phosflow Perm/Wahs Buffer I (BD Biosciences), and stained with CD49f–BV510 (BD Biosciences), CD29–APCe780 (Thermo Fisher Scientific), CD44–BB700 (BD Biosciences), pAKTs473–BV421 (BD Biosciences) and pJAK2y1007/1008–Alexa647 (Abcam) antibodies for 2 h on ice. The samples were then washed and analysed on the BD Biosciences LSR Fortessa system. The flow cytometry data were analysed using FlowJo (BD Biosciences).

### RNA purification and ATAC-seq library preparation

For bulk RNA-seq, targeted cell populations from 2 (SCC) to 15 (papilloma) tumours per population were directly sorted into TRI Reagent (Thermo Fisher Scientific) and the total RNA was purified using the Direct-zol RNA MiniPrep Kit (Zymo Research) according to the manufacturer’s instructions. The integrity of purified RNA was determined using the Agilent 2100 Bioanalyzer. Library preparation, using the Illumina TrueSeq mRNA sample preparation kit (non-stranded, poly(A) selection), and sequencing were performed at the Genomic Core Facility at Weill Cornell Medical College on the Illumina HiSeq 4000 system with the 50 bp single-end setting or the NovaSeq with the 100 bp paired-end setting.

For accessible chromatin profiling, target cell populations from 2 (SCC) to 15 (papilloma) tumours per population were sorted into FACS buffer, and ATAC-seq sample preparation was performed as described previously^51^. In brief, a minimum of 2 × 10^4^ cells were lysed with ATAC lysis buffer on ice for 1 min. Lysed cells were then tagmented with Tn5 transposase (Illumina) at 37 °C for 30 min. Cleaned-up fragments were PCR-amplified (NEB) and size-selected with 1.8× SPRI beads (Beckman Coulter). Libraries were sequenced at the Genome Resource Center at The Rockefeller University on the Illumina NextSeq system with the 40 bp paired-end setting.

For scRNA-seq, target cell populations were sorted from 3–5 SCC tumours per mouse, for a total of 3 biological replicates (2 male and 1 female mice). Single-cell libraries were prepared according to a slightly modified Smart-seq2 protocol^52^. In brief, cells were sorted into 96-well plates containing hypotonic lysis buffer, snap-frozen with liquid nitrogen and stored at −80 °C until further processing. To semi-quantitatively assess technical variation between cells, ERCC spike-ins (1:2 × 10^6^ dilution, Thermo Fisher Scientific) were added with the lysis buffer. After thawing, cells were lysed at 72 °C for 3 min. Released RNA was reverse-transcribed using dT30 oligos, template switching oligos and Maxima H- reverse transcriptase. cDNA was amplified by 15 cycles of whole-transcriptome amplification using KAPA HiFi DNA polymerase (Roche) and then size-selected using 0.6× AmpPure XP beads (Beckman Coulter). To exclude wells containing multiple cells, as well as low-quality and empty wells, quantitative PCR with reverse transcription (RT–qPCR) for *Gapdh* was performed before proceeding. Illumina sequencing libraries were then prepared using the Nextera XT DNA library preparation kit (Illumina) and indexed with unique 5′ and 3′ barcode combinations. After barcoding, the samples were pooled and size-selected with 0.9× AmpPure XP beads. The integrity of the pooled library was assessed using the TapeStation (Agilent) before sequencing on two lanes of the Illumina NovaSeq S1 system using 100 bp paired-end read output (Illumina). For optimal sequencing depth, each sequencing library was sequenced twice, once in each lane of the Illumina NovaSeq system. Sequencing reads per cell from each lane were combined during alignment to the reference genome.

### CRISPR-mediated *Lepr* knockout

Our *Lepr*^*null*^ PDVC57 cell line was generated using the Alt-R CRISPR–Cas9 system (IDT). In brief, a recombinant Cas9 protein, validated sgRNA (GAGUCAUCGGUUGUGUUCGG) targeting exon 3 of the mouse *Lepr* gene or a negative control sgRNA (IDT), and an ATTO-550-conjugated tracer RNA were used to form a ribonucleoprotein (RNP) were mixed with RNAiMax reagent (Thermo Fisher Scientific). PDVC57 cells were then transfected with the mixture overnight and FACS-purified into 96-well plates to produce clonal cell lines. The *Lepr*^*null*^ PDVC57 cell line was selected after validating by immunoblot analysis of LEPR as well as sequencing of the target region for indel efficiency using the MiSeq system. The *Lepr*^*null*^ PDVC57 cell line and *Lepr*^*ctrl*^ PDVC57 cell line were intradermally injected into the immunocompromised Nude mice, and the tumours were analysed for growth and progression.

### Rescue of LEPR in *Lepr*^*null*^ PDVC57 SCC cells

*Lepr*^*null*^ PDVC57 cells were transduced in vitro with 1:1 ratio of PGK-rtTA3 lentivirus and *TRE-FL-Lepr-IRES-eGFP* or *TRE-Lepr*^*ΔSig*^*-IRES-eGFP* lentivirus. After culturing in 1 μg ml^−1^ of doxycycline (Sigma-Aldrich) containing E-Low medium, eGFP^high^ cells expressing *Lepr* were isolated by FACS and expanded in vitro. These two different cell lines were later intradermally grafted onto immunocompromised Nude mice, and the tumours were analysed for growth and progression.

### Limiting dilution assay

To compare the tumour-initiating ability between *Lepr*^*null*^ PDVC57 and *Lepr*^*ctrl*^ PDVC57 cell lines, a preset number of cells were intradermally grafted onto Nude mice, and the tumour growth was tracked for 5 weeks to calculate the tumorigenicity of cells. As previously described^23^, SCC cells were diluted serially from 10^4^ to 10^6^ cells per ml and 100 µl cell mixtures in 1:1 PBS:Matrigel were injected. Four injections per mouse were performed under the animal facility regulations (for 10^5^ and 10^4^ per injection, *n* = 4; for 10^3^ per injection, *n* = 8). Photos of mice were recorded, and tumours were counted at the end point 5 weeks after injection.

### Osmotic pump for systemic delivery

To achieve continuous systemic delivery of compounds, Alzet osmotic pumps were implanted as previously described^53^ into the back skins of Nude mice. Three weeks after the initial intradermal tumour grafts, tumour-bearing Nude mice were anaesthetized and sterilized for surgical procedures. A small cut was created with scissors and the osmotic pump containing a predetermined concentration of compounds or vehicle was inserted underneath the back skin and the opening was clipped. For the leptin experiment, 4-week-long delivery pumps were used with 2 mg ml^−1^ leptin (R&D Systems), 0.5 mg ml^−1^ leptin, 0.5 mg ml^−1^ SMLA (BioSources) and PBS vehicle. Where indicated, fluorescently labelled (680RD) leptin as previously discribed^54^ was used to detect the ability of circulating leptin to reach the skin stroma. For the VEGFA experiment, 4-week-long delivery pumps were used with 50 μg ml^−1^ VEGFA (R&D Systems) and PBS vehicle. For the rapamycin experiment, 2-week-long delivery pumps were used with 10 mM rapamycin (SelleckChem) in PBS solution with 10% DMSO or with the respective vehicle control. Tumour sizes were then monitored for tumour growth and progression.

To achieve local delivery of compound, intradermal injections were performed into the skin adjacent to or underneath the grafted tumours of Nude mice. 50 μg ml^−1^ VEGFA (R&D Systems) and PBS vehicle were injected in a 50 μl volume every 3 days with a 1 ml syringe and a 26G needle (BD Biosciences).

### RT–qPCR

To measure *Lep* mRNA levels in whole tissues, normal skin from wild-type FVB/N mice was first separated from the underlying white fat tissue, and then both were independently snap-frozen in liquid nitrogen. Papilloma and SCC tumours were trimmed from the adjacent aphenotypic skin. Frozen tissues were crushed and then dissolved in Tri-Reagent (Thermo Fisher Scientific). RNA was isolated using the Direct-zol RNA miniprep kit (Zymo Research). To measure *Lep* levels in specific cells from the tumour or normal microenvironment, CD45^+^ (immune cells), CD140a^+^ (fibroblasts and other mesenchymal cells), CD117^+^ (melanocytes) and CD31^+^ (endothelial cells) were FACS-isolated from single-cell suspensions of normal skin, papilloma and SCC in Tri-Reagent (Thermo Fisher Scientific). RNA was isolated using the Direct-zol RNA microprep kit (Zymo Research). Equivalent amounts of RNA were reverse-transcribed using the SuperScript VILO cDNA Synthesis Kit (Thermo Fisher Scientific). cDNAs were mixed with the primers listed below and the Power SYBR Green PCR Master Mix (Thermo Fisher Scientific), and then quantified using the Applied Biosystems QuantStudio 6 Real-Time PCR system. *Lep* levels were normalized to equal amounts using primers against *B2m*. Primer sequences were as follows: *Lep* forward, 5′-GAGACCCCTGTGTCGGTTC-3′; *Lep* reverse, 5′- CTGCGTGTGTGAAATGTCATTG-3′; *B2m* forward, 5′- TTCTGGTGCTTGTCTCACTGA-3′; *B2m* reverse, 5′-CAGTATGTTCGGCTTCCCATTC-3′.

### PI3K inhibitor gavage in tumour-bearing mice

As previously described^33^, the pan-PI3K inhibitor BKM120 (MedChemExpress) was dissolved in DMSO to 100 mg ml^−1^. A 10% (v/v) solution was then sequentially diluted in 40% PEG300, 5% Tween-80 and 45% PBS. DMSO (10%) was used as vehicle control. The course of treatment was daily gavage for 14 days. Tumour-bearing mice were first anaesthetized lightly and 100 µl of the solution was delivered to the mouse stomach through a feeding needle (Thermo Fisher Scientific). The study was blinded by one experimentalist performing gavage and the other one measuring the tumour sizes every 2–3 days without knowing the treatment or control. The results were analysed at day 15 after the initial treatment.

### Colony-forming assay

After LPER^+^ and LEPR^−^ tumour basal cells (CD29/CD49f^high^CD44^+^) were FACS isolated and counted, 5 × 10^4^ cells of each replicate per condition were plated in a 10 cm dish with a growth-inhibited 3T3/J2 feeder layer with the SY medium (E.F.’s laboratory, see below) at 7.5% CO_2_ and 37 °C. After 14 days, the cultures were fixed and stained with Alexa647-conjugated CD49f antibodies (BioLegend). The plates were then imaged using the LiCor Odyssey Imager and quantified on the basis of the numbers and sizes of colonies.

### SY mouse skin stem cell culture medium

The base medium was made with calcium-free DMEM/F12 (3:1) (E.F.’s laboratory) with 1× Glutamax (Thermo Fisher Scientific) and 1× penicillin–streptomycin (Thermo Fisher Scientific). Additives included 15% chelated fetal bovine serum (Thermo Fisher Scientific), 418.5 ng ml^−1^ of hydrocortisone (Sigma-Aldrich), 9.405 ng ml^−1^ of cholera toxin (Sigma-Aldrich), 10 μM of Y-27632 (Selleck Chemicals), 0.0525 mg ml^−1^ insulin (Sigma-Aldrich), 0.0525 mg ml^−1^ Apo-transferrin (Thermo Fisher Scientific), 300 mM CaCl_2_ (Sigma-Aldrich), 36.5 mM of NaHCO_3_ (Sigma-Aldrich) and 2.1 × 10^−8^ M of 3,3′,5-triiodo-l-thyronine (Sigma-Aldrich).

### Mouse leptin ELISA

For quantification of leptin level in the tissue or plasma, the Quantikine Mouse/Rat Leptin ELISA (R&D Systems) kits were used. Tumour tissues were snap-frozen without adjacent skin in liquid nitrogen and sonicated in lysis buffer (R&D Systems) before centrifuging at full speed at 4 °C for 10 min to obtain total lysates. Plasma was obtained by centrifuging clean blood for 15 min at 2,000*g* at 4 °C. The manufacture’s protocol was followed for these assays.

### Fluorescence assay for detecting labelled proteins

For quantification of 680RD-labelled leptin level in tissue, the Biotek Cytation 5 System (BioTek) was used. Tumour and skin tissues were snap-frozen in liquid nitrogen and sonicated in Lysis Buffer (R&D Systems) before centrifuging at full speed at 4 °C for 10 min to obtain total lysates. Serial dilutions of labelled recombinant protein were used as standards to generate a curve to estimate the amount of protein in the tissue lysates. A total of 100 μl of lysates and standards in duplicates were loaded into 96-well black assay plate (Thermo Fisher Scientific) and then read at an excitation of 680 nm and emission of 695 nm. Estimated concentrations were then calculated.

### Immunoblotting

To collect cells, cultured cells were washed on the plate in cold 1× PBS, lysed in RIPA Buffer (Millipore) supplemented with protease and phosphatase inhibitors (Roche), and collected by scraping. Cells were lysed for 30 min on ice and then centrifuged to collect the supernatant. The protein concentration was determined by the BCA assay (Pierce) against a bovine serum albumin standard curve. Protein (20 μg) of each sample was run on NuPAGE 4–12% Bis-Tris Gels (Invitrogen) for 1 h at 200 V in NuPAGE MES SDS Running Buffer (Invitrogen). Protein was transferred overnight onto the Immunoblon FL PVDF membrane (Millipore) in NuPAGE transfer buffer (Invitrogen) with methanol at 15 V and 4 °C. Membranes were blocked in Odyssey TBS blocking buffer for at least 1 h at room temperature before incubating with primary antibodies overnight at 4 °C in Odyssey buffer with Tween-20. Membranes were washed several times in 0.1% Tween-20 in PBS before incubating with fluorescent secondary antibody.

The following primary antibodies and dilutions were used: primary antibodies (anti-mLEPR 1:1,000, R&D Systems; anti-AKT, 1:1,000, Cell Signaling; anti-pAKT(S473), 1:1,000, Cell Signalling; anti-S6, 1:1,000, Cell Signaling; anti-pS6(S240/244), 1:1,000, Cell Signaling; anti-S6K, 1:1,000, R&D Systems; anti-pS6K(T389), 1:1,000, Cell Signaling; anti-GAPDH, 1:5,000, Thermo Fisher Scientific; anti-α-tubulin, 1:5,000, Sigma-Aldrich), secondary antibodies were used at 1:10,000 (donkey anti-rabbit HRP and donkey anti-mouse Alexa647, Jackson ImmunoResearch). Membranes were imaged with an GE Amsham AI600 Imager. Owing to multiple targeted proteins in each experiment, one set of identical samples with the same sample volumes and processing procedure was blotted for GAPDH or α-tubulin in one of the gels in the same experiment as a loading control.

### Bulk RNA-seq analysis

Trimmed fastq files were obtained from the Genomic Core Facility of Weill Cornell Medical College. The analysis was performed by the cluster at the High-Performance Computing facility. For RNA-seq analysis of C57BL/6J *TRE-HRAS*^*G12V*^ driven papilloma and SCC samples (Fig. 1 and Extended Data Fig. 1), raw sequencing reads were aligned to the mouse reference genome (UCSC release mm10) using Bowtie2 (v.2.2.9)^55^ using the default parameters. The expression values of each gene were quantified as transcripts per million (TPM), as well as raw counts, using RSEM (v.1.2.30)^56^. Differential gene expression analysis was performed on raw counts using DESeq2 (v.1.24.0) with a negative binomial distribution and Wald test for significance^57^. Genes with average counts of greater than 10, log_2_[fold change] > |1| and adjusted *P* < 0.05 were considered to be differentially expressed. Differentially expressed genes were presented as a heat map with *z*-score-normalized expression values. To examine temporal changes in regulators of angiogenesis as cells transit from normal to benign to invasive states, the expressed genes related to the GO term ‘positive regulation of angiogenesis’ (GO:0045766, AmiGO2) were plotted as a *z*-score-normalized heat map.

For RNA-seq analysis of FVB *TRE-HRAS*^*G12V*^-driven TGFβ-reporter papilloma and SCC samples, genome indices were generated with the genome sequence (GRCm38.p5) and the comprehensive gene annotation on the primary assembly (GENCODE M16). Raw reads were aligned to the genome indices and gene counts were generated using STAR (v.2.6)^58^ with the default parameters. For differential gene expression analysis, low-expressed genes (minimum average read count < 10) were filtered out before DESeq2 analysis (v.1.16.1) in R Studio (v.3.4.2). Paired mCherry-positive and -negative samples were identified as batches and disease stages (papilloma versus SCC) as conditions for differential gene expression modelling using a negative binomial distribution and Wald test by DESeq2. Genes were considered to be differentially expressed when log_2_[fold-change] > |1| and adjusted *P* < 0.05.

For PDV-WT and *Lepr*^*KO*^ grafted SCC samples, raw reads were mapped to the decoy-aware mouse genome (UCSC release mm10) using Salmon (v.1.4.0)^59^. The expression level of each gene was quantified as TPM, as well as by raw counts, using Tximport (v.1.12.3)^60^ in R (v.3.6.1). For differential gene expression analysis, low-expressed genes (minimum average read count < 10) were filtered out before DESeq2 analysis (v.1.16.1) in R Studio (v.3.4.2). Differential gene expression modelling used a negative binomial distribution and Wald test by DESeq2. Genes were considered to be differentially expressed for log_2_[fold-change] > |1| and adjusted *P* < 0.05.

GO term and KEGG pathway analysis were performed using the DAVID online tool (NIH).

### Bulk ATAC-seq analysis

Fastq files were obtained from the Genomic Resource Center of The Rockefeller University. The analysis was performed on the computational cluster at the High-Performance Computing facility. The raw sequencing reads were aligned using Bowtie2 (v.2.2.9)^55^ to the mm10 reference genome (UCSC). The aligned reads were de-duplicated with Picard (v.2.3.0; Broad Institute, 2019) and shifted to correct for Tn5 insertion bias. Peaks were called using MACS2 (v.2.1.1) with the default settings^61^. Next, all peaks from IFE, HFSC, PAPneg, PAPpos, SCCneg and SCCpos were concatenated to a union peak set, and the read coverage of each sample at these peaks was calculated with Bedtools (v.2.25). The R (v.3.6.1) package pheatmap (v.1.0.12) was then used to generate the heat map. For motif analysis, HOMER findMotifGenome.pl was used with a customized motif database from JASPAR 2018. The motif input for HOMER (v.4.11)^62^ was generated from JASPAR 2018 vertebrates CORE central motifs using 80% of the maximum log-odds expectation for each motif as the detection threshold for HOMER.

### Single-cell RNA-seq analysis

Sequence and transcript coordinates for the mouse release M23 (GRCm38.p6) genome and gene models were downloaded from GENCODE. Paired sequencing reads for scRNA-seq libraries were aligned to the mouse reference genome, combined with sequences for ERCC spike-ins as artificial chromosomes, using STAR (v.2.5.2a)^58^ with the default parameters for paired-end reads. Transcript expression values were calculated using the Salmon quantification software (v.0.14.1)^59^ and gene expression levels as TPMs and counts were obtained using Tximport (v.1.12.3)^60^. TPMs were transformed to log_2_[TPM + 1]. For downstream analyses, cells with <2,500 genes detected per cell and genes expressed in <5% of the cell population were removed. Cells expressing lower levels of the lineage marker *Krt14* (log_2_[TPM + 1] < 7) were excluded. After filtering, there were 1,504 cells (159 integrin^low^ suprabasal, 500 integrin^high^, mCherry^−^ basal, and 845 integrin^high^, mCherry^+^ basal cells) (*n* = 3 mice) in the dataset.

Analyses and visualization of data were conducted in a Python environment built on the Numpy, SciPy, matplotlib, scikit-learn package and pandas libraries. To distinguish true biological variability in gene expression from technical noise, we used a statistical model for identifying highly variable genes compared to ERCC spike-ins as described^63^. In brief, we used a custom script based on the methodology described^63^, running in R v.3.6.1, to identify those genes with a higher level of variation (at least 10% above the technical variation) and a false-discovery rate (FDR) value of less than 0.1. To identify cell clusters and visualize the data, we first centred and scaled the highly variable gene dataset and performed principal component analysis on the list of highly variable genes. The first 201 principal components, which captured 50% of the variance in the dataset, were used as an input for nonlinear dimensionality reduction, performed using UMAP implemented in scikit-learn. To identify clusters, we used a graph-based clustering approach based on building a *k*-nearest neighbours graph and clustering with the Louvain algorithm (with *k* set to one fifth of the dataset size, and a resolution parameter of 1 × 10^−4^). Euclidean distance in PCA space served as input for both UMAP generation and Louvain clustering.

Differential gene expression was used to identify genes specific to each cluster. In brief, we used raw count matrices for expressed genes and applied them to the DESeq2 package (v.1.24.0)^57^ using R. We used a negative binomial fit to model differential gene expression, factored the dataset based on the Louvain cluster assignments, and used a threshold of 0.75 to construct Wald tests of significance. Genes were considered to be differentially expressed if log_2_[fold change] > |1| and adjusted *P* < 0.05. Low-expressed differential genes (baseMean expression < 200) were discarded from visualization and further analysis. The expression levels of specific genes of interest were visualized as log_2_[TPM + 1] values on the corresponding UMAP representation of the data. GO term and KEGG pathway analyses were performed using the DAVID online tool (NIH).

To generate comprehensive gene set scores based on GO term analyses, for example, for angiogenesis or AKT signalling pathways, the corresponding *Mus musculus* gene lists were obtained from AmiGO 2 through the Gene Ontology consortium. The AddModuleScore function of Seurat (v.3.1.1) was used to calculate the average expression levels of each gene set at the single-cell level, subtracted by the aggregated expression of control feature sets, as originally described^64^. The resulting gene set scores for each cell were colour coded on corresponding UMAP visualizations of the data.

### Statistics and reproducibility

For the mouse experiments, group sizes were determined by power analysis using preliminary experimental data. All of the experimental measurements were taken from independent distinct samples. Unless stated otherwise, statistical analysis was performed using unpaired two-tailed Student’s *t*-tests with a 95% confidence interval under the untested assumption of normality on the GraphPad Prism (v.9.0). All of the error bars in the box plots and growth curves are mean ± s.e.m. The degree of statistical significance is denoted by asterisks; NS, *P* ≥ 0.05; *P < 0.05, **P < 0.01, ***P < 0.001, ****P < 0.0001. Whenever representative plots or images are shown, the datasets with similar results were generally generated from more than one litter of mice and with *n* ≥ 3 independent biological replicates, due to the nature of the tumour staging being histopathology driven rather than age or size driven. All attempts at replication in this study were successful. In general, the experiments were not randomized or performed by the investigator in a blinded manner, except where stated.

### Schematics

Schematics were prepared using Office 365 (Microsoft), BioRender (www.biorender.com) with publication permissions and Affinity Design (Serif Europe).

### Reporting summary

Further information on research design is available in the Nature Portfolio Reporting Summary linked to this article.

## Online content

Any methods, additional references, Nature Portfolio reporting summaries, source data, extended data, supplementary information, acknowledgements, peer review information; details of author contributions and competing interests; and statements of data and code availability are available at 10.1038/s41586-022-05475-6.

### Supplementary information

Supplementary Information Supplementary Figs. 1–4 and the legends for Supplementary Tables 1–5.

Reporting Summary

Supplementary Table 1

Supplementary Table 2

Supplementary Table 3

Supplementary Table 4

Supplementary Table 5

## Data Availability

All data supporting the findings of this study are available within the Article and its Supplementary Information. All single-cell and bulk sequencing data generated within this study have been deposited at the Gene Expression Omnibus (GEO) under accession code GSE190415. Source data are provided with this paper.

## Source data

Source Data Fig. 1
Source Data Fig. 3
Source Data Fig. 4
Source Data Fig. 5
Source Data Extended Data Fig. 1
Source Data Extended Data Fig. 2
Source Data Extended Data Fig. 3
Source Data Extended Data Fig. 5
Source Data Extended Data Fig. 6
Source Data Extended Data Fig. 7
Source Data Extended Data Fig. 8

## Code availability

Custom code for scRNA-seq for this study has been deposited at Zenodo (10.5281/zenodo.7186350). All of the other codes are available from the corresponding author on reasonable request.
