# Supplementary material for: Ras drives malignancy through stem cell crosstalk with the microenvironment
Source: Nature. 2022 Nov 30;612(7940):555–63. doi: 10.1038/s41586-022-05475-6 (PMC9750880; doi:10.1038/s41586-022-05475-6)
Supplement: Supplementary file 1 — Supplementary Figs. 1–4 and the legends for Supplementary Tables 1–5. [file 41586_2022_5475_MOESM1_ESM.pdf]

nature portfolio

https://doi.org/10.1038/s41586-022-05475-6

Supplementary information

Ras drives malignancy through stem cell crosstalk with the microenvironment

In the format provided by the
authors and unedited

SI Guide

Ras drives malignancy through stem cell crosstalk with the microenvironment

Shaopeng Yuan[1], Katherine S. Stewart[1], Yihao Yang[1], Merve Deniz Abdusselamoglu[1], S. Martina Parigi[1], Tamar Y. Feinberg[1,2], Karen Tumaneng[1,3], Hanseul Yang[1,4], John M. Levorse[1,5], Lisa Polak[1], David Ng[1], Elaine Fuchs[1,6,*]

[1]Robin Chemers Neustein Laboratory of Mammalian Cell Biology and Development, The Rockefeller University, New York, NY 10065, USA

[2]Present address: Volastra Therapeutics, New York, NY 10027, USA

[3]Present address: Sanofi, Cambridge, MA 02141, USA

[4]Present address: Department of Biological Sciences, Korea Advanced Institute of Science and Technology, 34141, Korea

[5]Present address: Temple University, Philadelphia, PA 19122 USA

[6]Howard Hughes Medical Institute, New York, NY 10065, USA

*Corresponding author in print: fuchslb@rockefeller.edu

Correspondence with the editors during the review process: fuchs@rockefeller.edu

Table of Contents

SI Figure 1 | Uncropped immunoblots from Main Figures. a, Immunoblots for Fig. 2e showing that cultured $HRAS^{G12V}$ keratinocytes (KT) that are wild type (FF) but not mutant (ΔΔ) for the TGFβ receptor gene (*Tgfbr2*) elevate LEPR dramatically in response to active recombinant TGFβ1. GAPDH is used as the loading control. Due to the usage of fluorescent secondary antibodies, they were run on different gels with identical amounts (20μg) of the same processed samples. **b**, For Fig. 5d and 5g, immunoblots of proteins isolated from $Lepr^{null}$ and $Lepr^{Ctrl}$ SCC cells treated with recombinant leptin or vehicle control for 48 hr prior to analyses. They show that leptin-dependent activation of pAKT in $LEPR^{+}$ cells, as well as higher overall levels of AKT, so do pS6K and pS6 as the hallmark of mTOR signalling. . GAPDH is used as the loading control. Due to the large number of targets to analyse, they were run on different gels with identical amounts (20μg) of the same processed samples.

SI Figure 2 | Uncropped immunoblots from Extended Data Figures. a, Immunoblots for Extended Data Fig. 5b showing that $Lepr^{null}$ PDVC57 SCC cells (Clone 2) generated by targeted CRISPR/CAS9 technology have a complete loss of LEPR, which was selected for the study. GAPDH is used as the loading control. They were run on the same gel. **b**, Immunoblots for Extended Data Fig. 5d showing $Lepr^{null}$ SCC cells transduced with either *TRE-*$Lepr^{FL}$ or *TRE-*$Lepr^{\Delta Sig}$ are validated with pan-LEPR analyses. α-Tubulin is used as the loading control. They were run on the same gel.

SI Figure 3 | FACS gating strategies for SCC cells for scRNAseq. FACS strategies for isolating basal progenitors from papilloma and SCCs, respectively ($\alpha6^{hi}\beta1^{hi}CD44^{+}$) that are either high or negative for TGFβ reporter mCherry. Papillomas and SCCs were analysed according to timing and pre-screened for pathology prior to FACS.

SI Figure 4 | FACS gating strategies for normal and cancer basal cells. FACS strategies for isolating basal progenitors from (top) normal telogen-phase skin ($\alpha6^{hi}\beta1^{+}$), inclusive of interfollicular epidermal SCs, upper hair follicle and sebaceous gland SCs and bulge HFSCs; and from (middle and bottom) papilloma and SCCs, respectively ($\alpha6^{hi}\beta1^{+}CD44^{+}$). Papillomas and SCCs are analysed according to timing and prescreened for pathology prior to FACS. For high throughput RNA sequencing, two independent replicates of FACS-isolated cells are used for each condition. Note also that for all other experiments performed on FACS-purified cells, the gating stringency is raised to 5 x 10^3 for CD29 (β1) and CD49f (α6).

Supplementary Table 1 to 5 (uploaded as separate Excel files)

Supplementary Table 1: Significantly up-regulated genes in normal versus tumour basal progenitor cells (TPMs per Sample)

Supplementary Table 2: Significantly up-regulated genes in papilloma versus SCC basal progenitor cells (TPMs per Sample)

Supplementary Table 3: Genes enriched for cluster C2 expression (relative to clusters C1 and C3) as TPMs

Supplementary Table 4: Significantly Up-regulated CSC genes by TGFβ responding tumour basal cells versus TGFβ non-responding tumour basal cell (TPMs per Sample)

Supplementary Table 5: Significantly Up-regulated genes that are unique in SCC-CSC (TPMs per Sample)

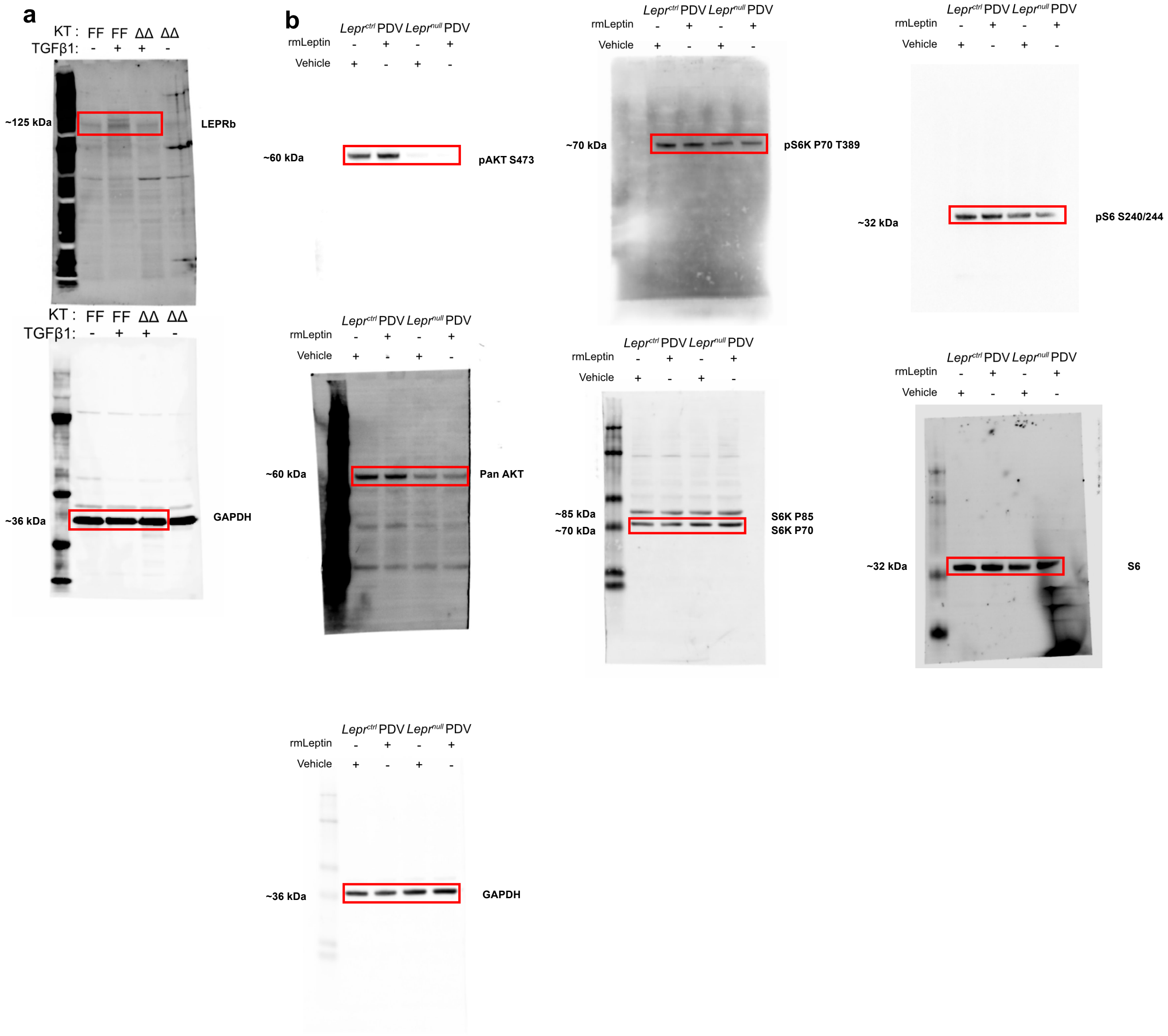

Supplementary Information Figure 1

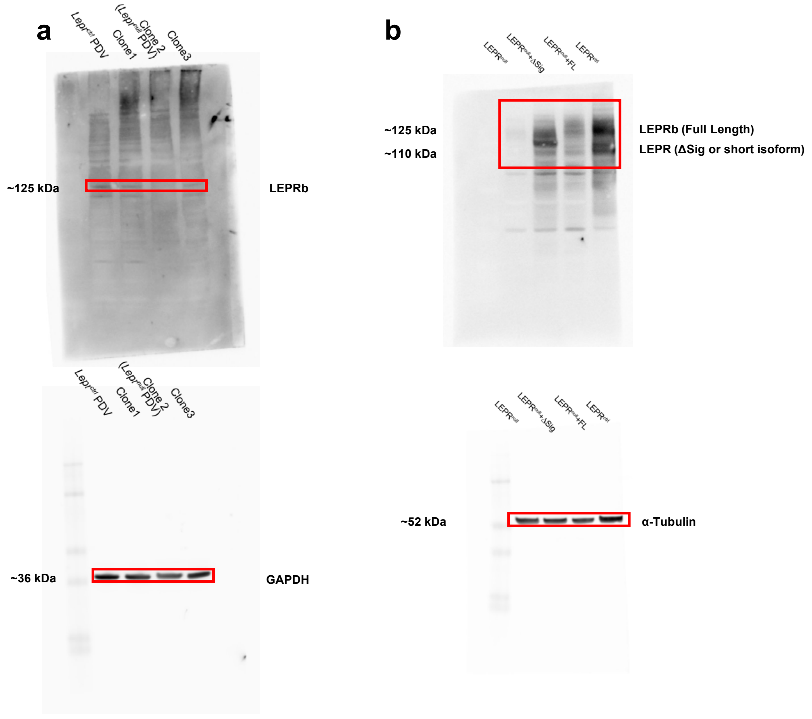

Supplementary Information Figure 2

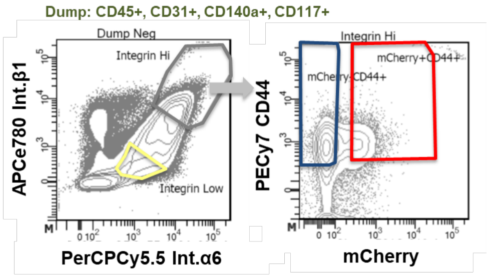

Supplementary Information Figure 3

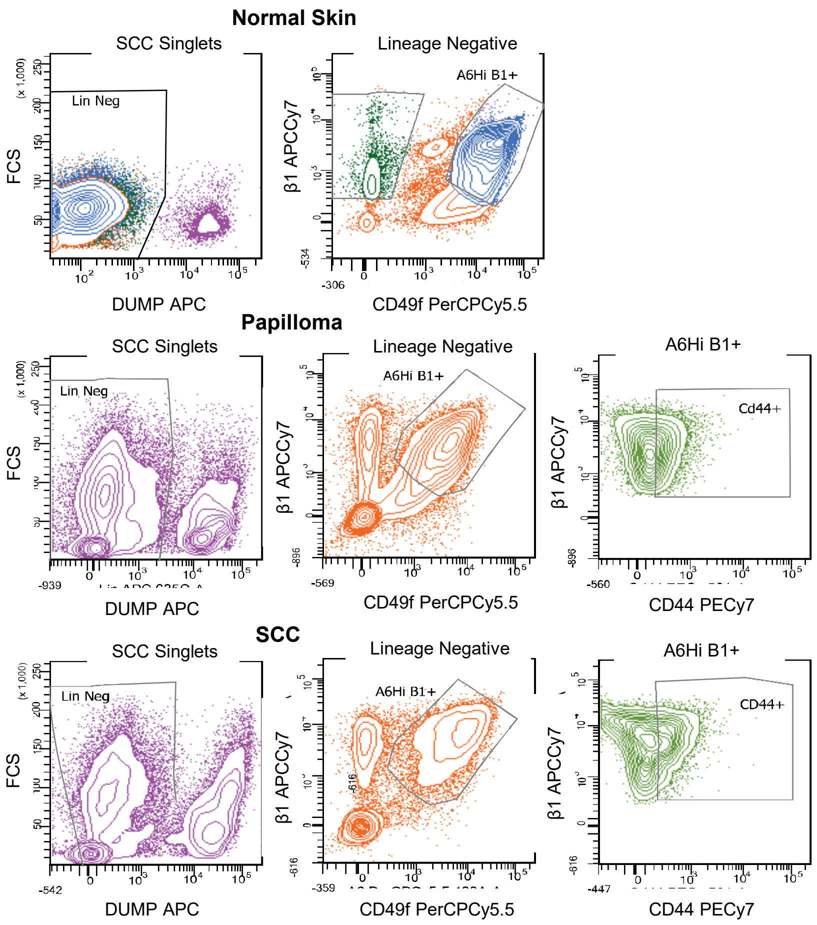

Supplementary Information Figure 4
