# Supplementary material for: Ras drives malignancy through stem cell crosstalk with the microenvironment
Source: Nature. 2022 Nov 30;612(7940):555–63. doi: 10.1038/s41586-022-05475-6 (PMC9750880; doi:10.1038/s41586-022-05475-6)
Supplement: Supplementary file 2 — Reporting Summary [file 41586_2022_5475_MOESM2_ESM.pdf]

nature portfolio

Corresponding author(s): Elaine Fuchs

Last updated by author(s): Oct 10, 2022



# Reporting Summary

Nature Portfolio wishes to improve the reproducibility of the work that we publish. This form provides structure for consistency and transparency in reporting. For further information on Nature Portfolio policies, see our Editorial Policies and the Editorial Policy Checklist.

## Statistics

For all statistical analyses, confirm that the following items are present in the figure legend, table legend, main text, or Methods section.

| n/a | Confirmed | |
|---|---|---|
| ☐ | ☒ | The exact sample size (*n*) for each experimental group/condition, given as a discrete number and unit of measurement |
| ☐ | ☒ | A statement on whether measurements were taken from distinct samples or whether the same sample was measured repeatedly |
| ☐ | ☒ | The statistical test(s) used AND whether they are one- or two-sided<br>*Only common tests should be described solely by name; describe more complex techniques in the Methods section.* |
| ☐ | ☒ | A description of all covariates tested |
| ☐ | ☒ | A description of any assumptions or corrections, such as tests of normality and adjustment for multiple comparisons |
| ☐ | ☒ | A full description of the statistical parameters including central tendency (e.g. means) or other basic estimates (e.g. regression coefficient) AND variation (e.g. standard deviation) or associated estimates of uncertainty (e.g. confidence intervals) |
| ☐ | ☒ | For null hypothesis testing, the test statistic (e.g. *F*, *t*, *r*) with confidence intervals, effect sizes, degrees of freedom and *P* value noted<br>*Give P values as exact values whenever suitable.* |
| ☒ | ☐ | For Bayesian analysis, information on the choice of priors and Markov chain Monte Carlo settings |
| ☒ | ☐ | For hierarchical and complex designs, identification of the appropriate level for tests and full reporting of outcomes |
| ☒ | ☐ | Estimates of effect sizes (e.g. Cohen's *d*, Pearson's *r*), indicating how they were calculated |

*Our web collection on statistics for biologists contains articles on many of the points above.*

## Software and code

Policy information about availability of computer code

| | |
|---|---|
| Data collection | BD FACSDiva (v. 8.0) for FACS sorting; G*Power (v3.1) for statistics; Bowtie2 (v. 2.2.9), RSEM (v. 1.2.30), STAR (v. 2.6 & 2.5.2a), DESeq2 (v. 1.16.1 & 1.24.0), R Studio (v. 3.4.2), Salmon (v. 0.14.1 & 1.4.0), Txîmport (v. 1.12.3), R (v. 3.6.1), Picard (v. 2.3.0), Bedtools (v. 2.25), Pheatmap (v. 1.0.12), HOMER (v. 4.11) for bulk RNA-seq, ATAC-seq, and scRNA-seq; Zen (v.3.1) for microscopy image collection; Image Studio (v 5.2) for image collection of colony forming assay; Amersham Imager 600 analysis (v 1.0) for western blot imaging. |
| Data analysis | Flowjo (v.9.0) for flowcytometry analysis; Graphpad Prism (v.9.0) for data analysis and plotting; Imaris (v9.5) for 3D image rendering and analysis. Custom code for scRNAseq for this study has been deposited in Zenodo, with accessible DOI: 10.5281/zenodo.7186350. All the other codes are available from the corresponding author upon reasonable request. |

For manuscripts utilizing custom algorithms or software that are central to the research but not yet described in published literature, software must be made available to editors and reviewers. We strongly encourage code deposition in a community repository (e.g. GitHub). See the Nature Portfolio guidelines for submitting code & software for further information.

## Data

Policy information about availability of data

All manuscripts must include a data availability statement. This statement should provide the following information, where applicable:

- Accession codes, unique identifiers, or web links for publicly available datasets
- A description of any restrictions on data availability
- For clinical datasets or third party data, please ensure that the statement adheres to our policy

All data that support the findings of this study are available within the paper and its supplementary files. All single-cell and bulk sequencing data generated within this study have been deposited in the Gene Expression Omnibus (GEO) repository with the accession code GSE190415. ATACseq and bulk RNAseq data were aligned

to mm10 reference genome (UCSC); scRNAseq data were aligned to GENCODE release M23 (GRCm38.p6).
mm10 UCSC: https://hgdownload.soe.ucsc.edu/;goldenPath/mm10/bigZips/
M23 Gencode: https://www.gencodegenes.org/mouse/release_M23.html

# Field-specific reporting

Please select the one below that is the best fit for your research. If you are not sure, read the appropriate sections before making your selection.

☒ Life sciences ☐ Behavioural & social sciences ☐ Ecological, evolutionary & environmental sciences

For a reference copy of the document with all sections, see nature.com/documents/nr-reporting-summary-flat.pdf

# Life sciences study design

All studies must disclose on these points even when the disclosure is negative.

**Sample size**

For mouse tumour growth experiments, group sizes were determined by the t-test based a priori power analysis with preliminary experimental data (G*Power v3.1) For the simple comparison of Lepr ctrl and null grafted tumors, n=4 were used. For the complex grafted tumor experiments, minimum n=6 were used. No sample size calculation was performed for the other experiments. All the exact sample size were stated in the figure legends or the methods section.

**Data exclusions**

No data was excluded from analysis

**Replication**

For bulk RNA-seq and ATAC-seq from the Hras tumor model, targeted cell populations from 2 (SCC) -15 (Papilloma) tumors per condition were isolated from two distinct replicates. For bulk RNA-seq from the grafted tumor model, targeted cell populations per condition were isolated from four replicates. For scRNA-seq, targeted cell populations were isolated from three litter-mates in three separated FACS experiments and checked reproducibility. The core experiment of Lepr null vs Lepr ctrl tumour growth has essentially been repeated as the vehicle conditions in Fig. 5e,j. All attempts at replication in this study were successful.

**Randomization**

For the Hras tumour model, there is no randomization needed, since the choose of samples were purely pathology driven after tumorigenesis and sample processing. For grafted tumor model, the tumor cells were injected to randomized female Nude litter-mates, and the same mouse always carried null and ctrl tumors. For drug treatment experiments, it was also randomized which mice getting the treatment or vehicle control. In general, the experiments were not randomized nor blinded to the investigator, except where stated. Since the temporal and spontaneous nature of our models, the investigator would not able to decide which mouse get papilloma or SCC. Most of the tumour experiments were pathology driven, which means post-experimental staging will decide the experiment groups the samples belong to.

**Blinding**

Due to the extensive handling during the PI3K inhibitor gavage experiment, the study was blinded by one experimentalist performing gavage daily and the other one measuring the tumor sizes every 2-3 days without knowing the treatment or control. In general, the experiments were not randomized nor blinded to the investigator, except where stated. Additionally, the investigator will able to see the skin cancer changes. However, most of the tumour experiments were pathology driven, as the investigator would not be able surely staging the tumour before harvesting the tissue.

# Reporting for specific materials, systems and methods

We require information from authors about some types of materials, experimental systems and methods used in many studies. Here, indicate whether each material, system or method listed is relevant to your study. If you are not sure if a list item applies to your research, read the appropriate section before selecting a response.

## Materials & experimental systems

| n/a | Involved in the study | |
|---|---|---|
| ☐ | ☒ | Antibodies |
| ☐ | ☒ | Eukaryotic cell lines |
| ☒ | ☐ | Palaeontology and archaeology |
| ☐ | ☒ | Animals and other organisms |
| ☐ | ☒ | Human research participants |
| ☒ | ☐ | Clinical data |
| ☒ | ☐ | Dual use research of concern |

## Methods

| n/a | Involved in the study | |
|---|---|---|
| ☒ | ☐ | ChIP-seq |
| ☐ | ☒ | Flow cytometry |
| ☒ | ☐ | MRI-based neuroimaging |

## Antibodies

**Antibodies used**

PE/Cy7 anti-mouse CD117, rat monoclonal (Clone 2B8) Biolegend Cat#135112 Lot#B260697
BV510 anti-mouse CD45, rat monoclonal (Clone 30-F11) BD Bioscience Cat#563891 Lot#7293934
Alexa647 conjugated anti-pJAK2(Y1007/1008), rabbit monoclonal (Clone E132) Abcam Cat#ab200340 Lot#GR3376679-2
Anti-mCherry/RFP, guinea pig polyclonal Fuchs lab
Anti-Keratin5, guinea pig polyclonal Fuchs lab

Anti-Keratin8, rabbit polyclonal Fuchs Lab
Anti-Keratin14, chicken polyclonal Biolegend Cat#906001 Lot#B299611
Anti-Keratin18, rabbit polyclonal Fuchs Lab
APC conjugated anti-CD45, rat monoclonal (Clone 30-F11) Biolegend Cat#103112 Lot#B327333
APC conjugated anti-CD31, rat monoclonal (Clone 390) ThermoFisher Cat#17-0311-82 Lot#2193898
APC conjugated anti-CD117, rat monoclonal (Clone 2B8) Biolegend Cat#105812 Lot#B334363
APC conjugated anti-CD140a, rat monoclonal (Clone APA5) ThermoFisher Cat#17-1401-81 Lot#2279166
PE/Cy7 anti-mouse CD44, rat monoclonal (Clone IM7) BD Bioscience Cat#560569 Lot#1221629
BV421 anti-mouse CD44, rat monoclonal (Clone IM7) BD Bioscience Cat#563970 Lot#0027946
BV421 anti-mouse pAKT(S473), mouse monoclonal (Clone M89-61) BD Bioscience Cat#562599 Lot#1024172
FITC conjugated anti-CD45, rat monoclonal (Clone 30-F11) Biolegend Cat#103108 Lot#B304667
FITC conjugated anti-CD31, rat monoclonal (Clone 390) Biolegend Cat#102406 Lot#B355048
FITC conjugated anti-CD117, rat monoclonal (Clone 2B8) Biolegend Cat#105806 Lot#B270127
FITC conjugated anti-CD140a, rat monoclonal (Clone APA5) ThermoFisher Cat#11-1401-82 Lot#2410902
PE/Cy7 anti-mouse CD49f, rat monoclonal (Clone GoH3) eBioscience Cat#25-0495-82 Lot#E14405-107
APC/Cy7 anti-mouse CD29, hamster monoclonal (Clone HMb1-1) Biolegend Cat#102226 Lot#B210996
APC/eFluor780 anti-mouse CD29, hamster monoclonal (Clone HMb1-1) ThermoFisher Cat#47-0291-82 Lot#2297505
PerCP/Cy5.5 anti-mouse CD49f, rat monoclonal (Clone GoH3) Biolegend Cat#313617 Lot#B303128
Anti-CD31, Armenian hamster monoclonal (Clone 2H8) Sigma Cat#MAB1398Z
Anti-Integrin-a6/CD49f, rat monoclonal (Clone GoH3) BD Bioscience Cat#555734 Lot#1005046
Anti-Phospho-Smad2 (Ser465/467) Rabbit monoclonal(Clone 138D4) Cell Signaling Cat# 3108S Lot#10
Anti-GAPDH, mouse monoclonal (Clone 6C5) ThermoFisher Cat#AM4300 Lot#01062535
Anti-alpha tubulin, mouse monoclonal (Clone DM1A) Sigma Cat#T6199
Anti-human LEPR, rabbit polyclonal Sigma Cat#HPA030899
Biotin conjugated anti-mouse LEPR, goat polyclonal R&D Systems Cat#BAF497 Lot#BVF0820121
anti-p70 S6 Kinase, mouse monoclonal (Clone215247) R&D Systems Cat#MAB8962 Lot#IYI0220031
anti- p-p70 S6 Kinase (T389), rabbit monoclonal (CloneD68F8) Cell Signaling Cat#9234S Lot#12
anti-S6, rabbit monoclonal (Clone5G10) Cell Signaling Cat#2217S Lot#7
anti-p-S6 (S240/244), rabbit monoclonal (CloneD68F8) Cell Signaling Cat#5365T Lot#8
anti-AKT (pan), mouse monoclonal (40D4) Cell Signaling Cat#2920S Lot#8
anti-pAKT(S473), rabbit monoclonal (D9E) Cell Signaling Cat#4060S Lot#26
anti-cFos, rabbit monoclonal (Clone9F6) Cell Signaling Cat#2250S Lot#9

Validation

The mouse LEPR antibody from R&D Systems was validated by immunofluoresent staining as part of experiment design (Fig. 3c). The human LEPR antibody from Sigma was validated by Human Protein Altlas (https://www.sigmaaldrich.com/US/en/product/sigma/hpa030899). The fluorophore conjugated antibodies from BD, ThermoFisher and Biolegend were validated as stated in their websites (https://www.bdbiosciences.com/en-us/products/reagents/flow-cytometry-reagents/research-reagents/quality-and-reproducibility, https://www.thermofisher.com/us/en/home/life-science/antibodies.html, https://www.biolegend.com/en-us/kokd-validation). The signaling antibodies from Cell Signaling and R&D Systems were validated as stated in their websites (www.www.cellsignal.com, www.rndsystems.com). Antibodies produced in Fuchs Lab were previously described and validated.

# Eukaryotic cell lines

Policy information about cell lines

Cell line source(s)

human skin SCC line A431 was from ATCC; mouse skin SCC PDVC57 was a gift from the original laboratory created it; mouse keratinocyte cell lines FF (Tgfbr2f/f PGK-HRASG12V) and ΔΔ (Tgfbr2null PGK-HRASG12V) were generated in Fuchs Lab; mouse fibroblast 3T3/J2 has been passaged in the laboratory as feeders originated from the Laboratory of Howard Green; 293TN HEK cells were purchased from SBI directly as low passage (P2) for lentiviral packaging.

Authentication

PDVC57 was validated by karyotyping and grafting tests. Mouse keratinocyte cell lines were validated previously in Fuchs Lab. 3T3/J2 has been functionally and morphologically validated as feeder cells. 293TN HEK cells were functionally test as SV40 containing cells producing lentivirus. A431 was not authenticated.

Mycoplasma contamination

The particular cells in the manuscript were not tested for mycoplasma, but our laboratory periodically tests tissue culture facility for mycoplasma contamination in general.

Commonly misidentified lines
(See ICLAC register)

None of the lines used in the study were in the ICLAC database.

# Animals and other organisms

Policy information about studies involving animals; ARRIVE guidelines recommended for reporting animal research

Laboratory animals

The original TRE-HRASG12V mice have been backcrossed 10 generations each to a C57Bl/6J background and an FVB/N background. FVB/N TRE-HRASG12V mice were bred to FVB/N R26-LSL-YFP mice to create the TGFβ-reporter lineage-tracing model. For the Tgfbr2 cKO experiment, FR-LSL-HRASG12V;Tgfbr2 fl/fl; R26-LSL-YFP mice were crossed in-house. Wildtype FVB/N mice have been used for any experiments related to normal skin. For tumor transplantation experiments, 7-9 weeks old female Nude/Nude mice from Charles River were used. All other studies used a mix of male and female mice, which for the assays used here, behaved similarly. In general, the ages of mice were from 3 weeks to 3 months for all the strains, only depending on the tumour progression. The animals were maintained and bred under specific-pathogen-free conditions at the Comparative Bioscience Center (CBC) at The Rockefeller University, which is an Association for Assessment and Accreditation of Laboratory Animal Care (AALAC) – accredited facility. Adult animals were housed in a cage with a maximum of 5 mice unless specific requirements were needed. The light cycle was from 7am to

| | |
|---|---|
| | 7pm. The temperature of the animal rooms was 20-26 ºC; the humidity of the animal rooms was 30-70%. All mouse protocols were approved by the Institutional Animal Care and Use Committee (IACUC) at the Rockefeller University. |
| Wild animals | The study did not use wild animals. |
| Field-collected samples | The study did not contain field-collected samples. |
| Ethics oversight | And all the procedures were performed with the Institutional Animal Care and Use Committee (IACUC) at The Rockefeller University–approved protocols (20012-H and 20066-H). |

Note that full information on the approval of the study protocol must also be provided in the manuscript.

# Human research participants

Policy information about studies involving human research participants

| | |
|---|---|
| Population characteristics | We obtained and stained one normal skin tissue and three human skin SCC tissues from Weill Cornell Medical College. The age and gender information is not available. |
| Recruitment | The subjects were recruited at Weill Cornell Medical College, according to approved IRB protocol. The samples were unidentified as NIH and Federal/State regulations. The samples were selected based on diagnosis to demonstrate the existence of the marker protein, so there is no selection bias that impacts results. |
| Ethics oversight | They were in accordance with approved Institutional Review Broad (IRB) protocol (EFU-0529) from Rockefeller, Weill Cornell Medical College, and Memorial Sloan Kettering Cancer Center. |

Note that full information on the approval of the study protocol must also be provided in the manuscript.

# Flow Cytometry

## Plots

Confirm that:

☒ The axis labels state the marker and fluorochrome used (e.g. CD4-FITC).

☒ The axis scales are clearly visible. Include numbers along axes only for bottom left plot of group (a 'group' is an analysis of identical markers).

☒ All plots are contour plots with outliers or pseudocolor plots.

☒ A numerical value for number of cells or percentage (with statistics) is provided.

## Methodology

| | |
|---|---|
| Sample preparation | To sort the target tumor cell populations, tumors were first dissected from the skin and minced in 0.25% of collagenase (Sigma) in HBSS (Gibco) solution. The tissue pieces were incubated at 37ºC for 20 minutes in a shaker. After a wash with ice-cold PBS and samples were further digested into single cell suspension in 0.25% Trypsin/EDTA (Gibco) for 10 min at 37ºC. After neutralization with the FACS buffer (5%FCS, 10mM EDTA, 1mM HEPES in PBS), single-cell suspension was then centrifuged, resuspended, and strained before preparing for staining. A cocktail of Abs for surface markers at the predetermined concentrations was prepared in the FACS buffer with 100ng/ml DAPI. The samples were stained on ice for 30min and washed with FACS buffer, then to FACS or analysis. |
| Instrument | BD FACSAria equipped with FACSDiva software for sorting, BD FACS Fortessa with FACSDiva software for analysis |
| Software | FACSDiva 8.0 for operating the sorter or analyzer. Flowjo 9.0 for further analysis. |
| Cell population abundance | During the FACS panel designing, post-sort were performed and reached to the satisfied levels of enrichment. Due to limited cell number, the entire targeted cell populations were directly sorted into desired lysis buffer for RNA-seq or FACS buffer for ATAC-seq. |
| Gating strategy | To isolate mouse skin SCC tumor basal cells, we first gated on CD31, CD45, CD117, and CD140a negative and live population, then we gate on CD29 and CD49f (basal integrins) high population, then gate on CD44+ (with FMO) to eliminate potential normal epithelial contamination, and further markers were gated by histogram as high or low. See detailed gating strategies in Extended Data Figure 1, 2. To isolate skin and tumour stroma cells, we first gate on CD31+, then CD140a+, then CD45+, then CD117+ separately. |

☒ Tick this box to confirm that a figure exemplifying the gating strategy is provided in the Supplementary Information.
